# Genomic analysis of *Plasmodium vivax* describes patterns of connectivity and putative drivers of adaptation in Ethiopia

**DOI:** 10.1038/s41598-023-47889-w

**Published:** 2023-11-27

**Authors:** Alebachew Messele Kebede, Edwin Sutanto, Hidayat Trimarsanto, Ernest Diez Benavente, Mariana Barnes, Richard D. Pearson, Sasha V. Siegel, Berhanu Erko, Ashenafi Assefa, Sisay Getachew, Abraham Aseffa, Beyene Petros, Eugenia Lo, Rezika Mohammed, Daniel Yilma, Angela Rumaseb, Francois Nosten, Rintis Noviyanti, Julian C. Rayner, Dominic P. Kwiatkowski, Ric N. Price, Lemu Golassa, Sarah Auburn

**Affiliations:** 1 Aklilu Lemma Institute of Pathobiology, Addis Ababa University, Addis Ababa, Ethiopia; 2 Exeins Health Initiative, Jakarta, Indonesia; 3 Menzies School of Health Research and Charles Darwin University, Casuarina, PO Box 41096, Darwin, NT 0811 Australia; 4 Eijkman Institute for Molecular Biology, Jakarta, Indonesia; 5 Laboratory of Experimental Cardiology, Department of Cardiology, University Medical Center Utrecht, Utrecht, The Netherlands; 6 Wellcome Sanger Institute, Hinxton, UK; 7 Ethiopian Public Health Institute, Addis Ababa, Ethiopia; 8 School of Public Health, Addis Ababa University, Addis Ababa, Ethiopia; 9 Armauer Hansen Research Unit (AHRI), Addis Ababa, Ethiopia; 10 Addis Ababa University, Addis Ababa, Ethiopia; 11 Millipore Sigma (Bioreliance), Rockville, USA; 12 Department of Microbiology and Immunology, College of Medicine, Drexel University, Philadelphia, USA; 13 University of Gondar, Gondar, Ethiopia; 14 Jimma University Clinical Trial Unit, Department of Internal Medicine, Jimma University, Jimma, Ethiopia; 15 Shoklo Malaria Research Unit, Faculty of Tropical Medicine, Mahidol University, Mae Sot, Thailand; 16 Centre for Tropical Medicine and Global Health, Nuffield Department of Medicine, University of Oxford, Oxford, UK; 17 Cambridge Institute for Medical Research, University of Cambridge, Cambridge, UK; 18 Mahidol-Oxford Tropical Medicine Research Unit, Mahidol University, Bangkok, Thailand

**Keywords:** Parasite genomics, Malaria, Population genetics, Genetic variation

## Abstract

Ethiopia has the greatest burden of *Plasmodium vivax* in Africa, but little is known about the epidemiological landscape of parasites across the country. We analysed the genomic diversity of 137 *P. vivax* isolates collected nine Ethiopian districts from 2012 to 2016. Signatures of selection were detected by cross-country comparisons with isolates from Thailand (*n* = 104) and Indonesia (*n* = 111), representing regions with low and high chloroquine resistance respectively. 26% (35/137) of Ethiopian infections were polyclonal, and 48.5% (17/35) of these comprised highly related clones (within-host identity-by-descent > 25%), indicating frequent co-transmission and superinfection. Parasite gene flow between districts could not be explained entirely by geographic distance, with economic and cultural factors hypothesised to have an impact on connectivity. Amplification of the duffy binding protein gene (*pvdbp1*) was prevalent across all districts (16–75%). Cross-population haplotype homozygosity revealed positive selection in a region proximal to the putative chloroquine resistance transporter gene (*pvcrt-o*). An S25P variant in amino acid transporter 1 (*pvaat1*), whose homologue has recently been implicated in *P. falciparum* chloroquine resistance evolution, was prevalent in Ethiopia (96%) but not Thailand or Indonesia (35–53%). The genomic architecture in Ethiopia highlights circulating variants of potential public health concern in an endemic setting with evidence of stable transmission.

## Introduction

*Plasmodium vivax* is becoming the predominant cause of malaria in the Asia–Pacific and Americas. However there is growing evidence of hidden reservoirs of infection within the African continent, with the highest number of cases reported from the Horn of Africa, where Ethiopia contributes 6% of the global burden of vivax malaria cases and large majority of African vivax malaria^1,2^. These disturbing trends highlight major shortcomings in current approaches to survey *P. vivax*.

*P. vivax* causes substantial morbidity in Ethiopia, accounting for approximately 30% of local malaria^1^. In 2020, the country reported a three year high of > 260 thousand cases of malaria^1^, and these numbers do not accurately capture the even greater burden of asymptomatic *P. vivax* present as blood or liver stage infections^3^. To date, local public health interventions in Ethiopia have been largely focused on the control of *P. falciparum*, but these methods have less impact on reducing the *P. vivax* reservoir^4,5^. Several biological factors make *P. vivax* harder to control than *P. falciparum*, including its ability to form dormant liver stages which can reactivate causing blood-stage infections in the absence of transmission, and the rapid development of gametocyte stages that enhance parasite transmission and complicate infection tracking. *P. vivax* blood-stage infections also typically have low parasite density, creating challenges in detecting and correctly diagnosing infected individuals^6^. In Ethiopia chloroquine remains the first line antimalarial treatment for patients with *P. vivax* malaria, although there are reports that its clinical efficacy may be declining^7–15^. Reliable molecular markers of chloroquine resistance (CQR) in *P. vivax* are urgently need to support surveillance for resistance, but these have yet to be defined^16^. To confound matters further there is wide heterogeneity in malaria epidemiology across Ethiopia due to significant variation in ecology, climate, altitude, and associated receptivity to *Anopheline* vectors. These factors further complicate public health decision-making on the most effective intervention strategies for specific regions^17^.

Population genomic studies of *P. vivax* have significant potential to deepen our understanding of the biology and epidemiology of this species. Previous genomic analyses have identified novel candidates of resistance to antimalarial drugs and selective pressures, as well as revealing a broad spectrum of diversity and population structure in different geographic regions, that reflect underlying patterns of transmission and spread of the parasite^18–24^. A recent genomic epidemiology study of 24 Ethiopian *P. vivax* isolates revealed a high prevalence of copy number amplification of the parasite duffy binding protein (*pvdbp1*) gene^25^; representing either a potential response to the high prevalence of duffy negativity in the human population, or an adaptive immune evasion strategy^26^. Using comparative analyses of extended haplotypes between Ethiopia and each of Thailand and Indonesia, the study also found evidence suggestive of selection in a region upstream of the chloroquine resistance transporter gene (*pvcrt-o*), a candidate determinant of chloroquine resistance^25^. However, the small sample size and limited geographic distribution of those samples across Ethiopia limited the inferences that could be drawn from these signals. Another recent study in Africa has identified a new malaria chloroquine resistance candidate; the amino acid transporter 1 gene (*pfaat1*) was implicated in the evolution of chloroquine resistance in *P. falciparum*^27^. However, the *P. vivax* amino acid transporter 1 gene had not been explored in Ethiopia. Here, using 137 high quality genomes collected across nine districts, we provide a detailed exploration of the natural selection and population structure of *P. vivax* in Ethiopia to inform on potential public health challenges.

## Results

### High-quality *P. vivax* genomes from nine districts of Ethiopia

From a total of 159 Ethiopian *P. vivax* genomes present in the MalariaGEN Pv4.0 dataset, we selected 137 high-quality samples (86.2%), with high-quality defined as having < 15% missing genotyping calls at a set of 410,900 high-quality biallelic SNPs (Supplementary Data 1). To compare these genomes with *P. vivax* from other regions, we used MalariaGEN Pv4.0 data from Thailand and Papua Indonesia^25^. Using the high-quality biallelic SNP set, a set of high-quality Thai (*n* = 104) and Indonesian (*n* = 111) samples were selected; a similar approach was taken in an earlier small-scale study of 24 Ethiopian *P. vivax* genomes^25^.

**Figure 1:**
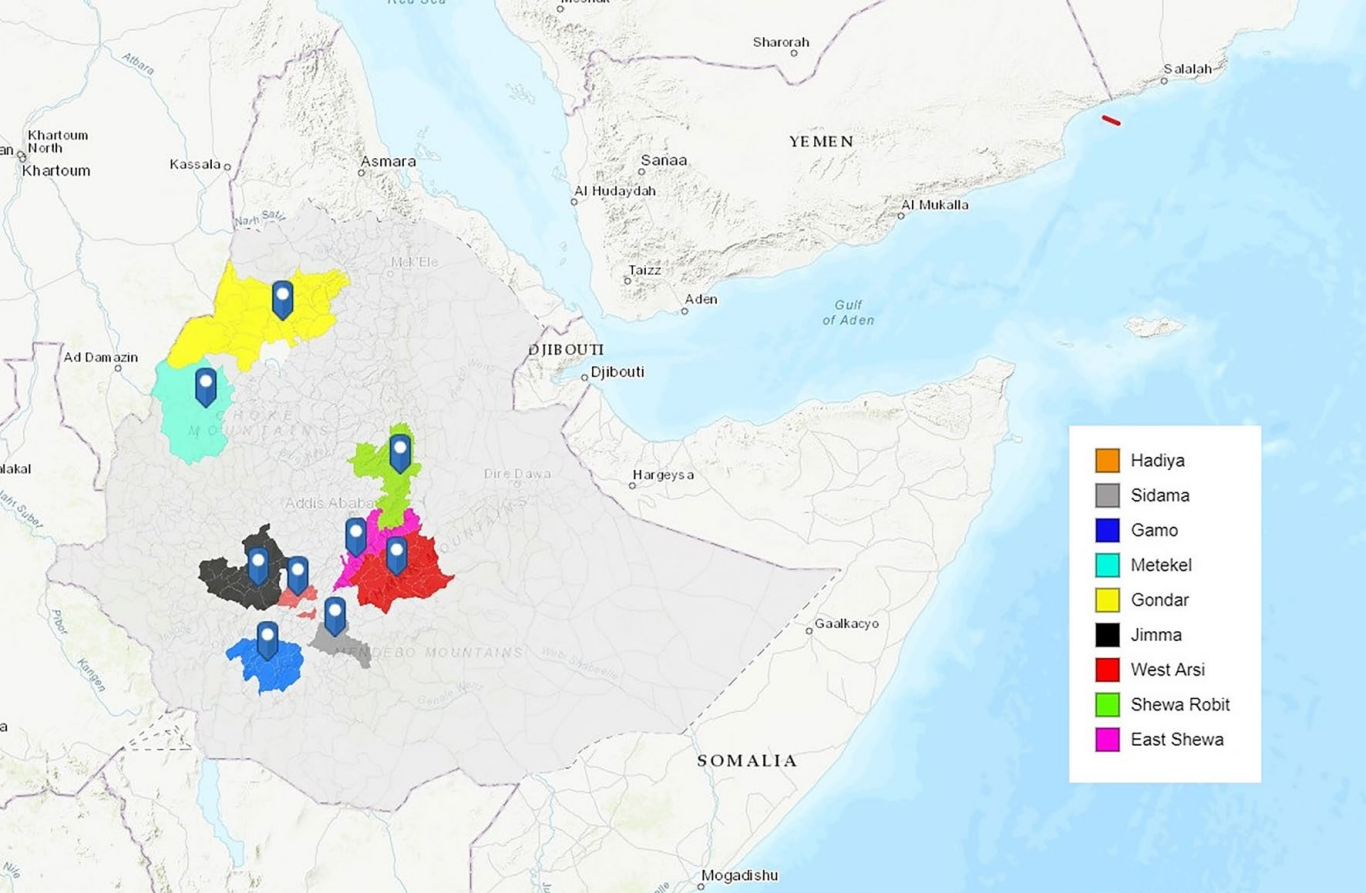
Study site locations. Map illustrating the locations of the nine study sites in Ethiopia, created using ArcGIS (http://www.arcgis.com, accessed September 2022).

The 137 Ethiopian samples included in the analysis were obtained from 10 districts, although one district (Badowacho) contributed only 1 isolate and therefore was not included in population-level analyses. The locations of the remaining nine districts are presented in Fig. 1, with malaria endemicity data summarised in (Supplementary Table 1). Although the small sample size (n < 10 isolates) from Gondar, Metekel, East Shewa, Sidama, Hadiya and Gamo suggest caution is needed in interpreting the data from these areas (Table 1), these districts were included in analyses to enable detection of geographic trends that could be evaluated further in future studies.

**Table 1:** Infection diversity and connectivity across districts.

District	Malaria endemicity^a^	*N*	% *F*_WS_ < 0.95	Median *F*_WS_ (range)	% Polyclonal infections with IBD ≥ 0.25 ^b^	% Polyclonal infections with IBD < 0.25 ^c^	Median interdistrict IBD (range)
Gondar	Moderate API > 10 and < 50	11	55% (6/11)	0.92 (0.58–1.00)	50% (3/6)	50% (3/6)	0.07 (0.06–0.08)
Metekel	Moderate/high: API ≥ 50	6	0% (0/6)	1.00 (0.99–1.00)	N/A	N/A	0.08 (0.06–0.09)
North Shewa	Very low: API < 5	17	12% (2/17)	1.00 (0.54–1.00)	0% (0/2)	100% (2/2)	0.05 (0.03–1)
East Shewa	Very low: API < 5	5	20% (1/5)	1.00 (0.91–1.00)	100% (1/1)	0% (0/1)	0.05 (0.03–0.09)
West Arsi	Very low: API < 5	48	25% (12/48)	1.00 (0.40–1.00)	50% (6/12)	50% (6/12)	0.07 (0.04–1)
Sidama	Very low: API < 5	9	33% (3/9)	0.99 (0.52–1.00)	33% (1/3)	67% (2/3)	0.1 (0.07–1)
Hadiya	Very low: API < 5	5	20% (1/5)	0.97 (0.67–1.00)	100% (1/1)	0% (0/1)	0.07 (0.06–0.08)
Jimma	Very low: API < 5	26	27% (7/26)	0.99 (0.54–1.00)	57% (4/7)	43% (3/7)	0.06 (0.01–0.17)
Gamo	Moderate: API/high > = 50	9	33% (3/9)	0.99 (0.30–1.00)	33% (1/3)	67% (2/3)	0.07 (0.05–0.1)

^a^Malaria endemicity defined by the National Malaria Elimination Program (NMEP) 2020 malaria stratification plan; API, Annual Parasite Incidence (malaria cases/ 1000 population). ^b^Percentage of polyclonal infections with IBD > = 0.25 in a minimum of two clones. ^c^Percentage of polyclonal infections with IBD < 0.25 in a minimum of two clones. ^bc^Note, infections comprising 3 or more clones can be represented in both categories.

### High frequency of superinfection and coinfection in Ethiopia

**Figure 2:**
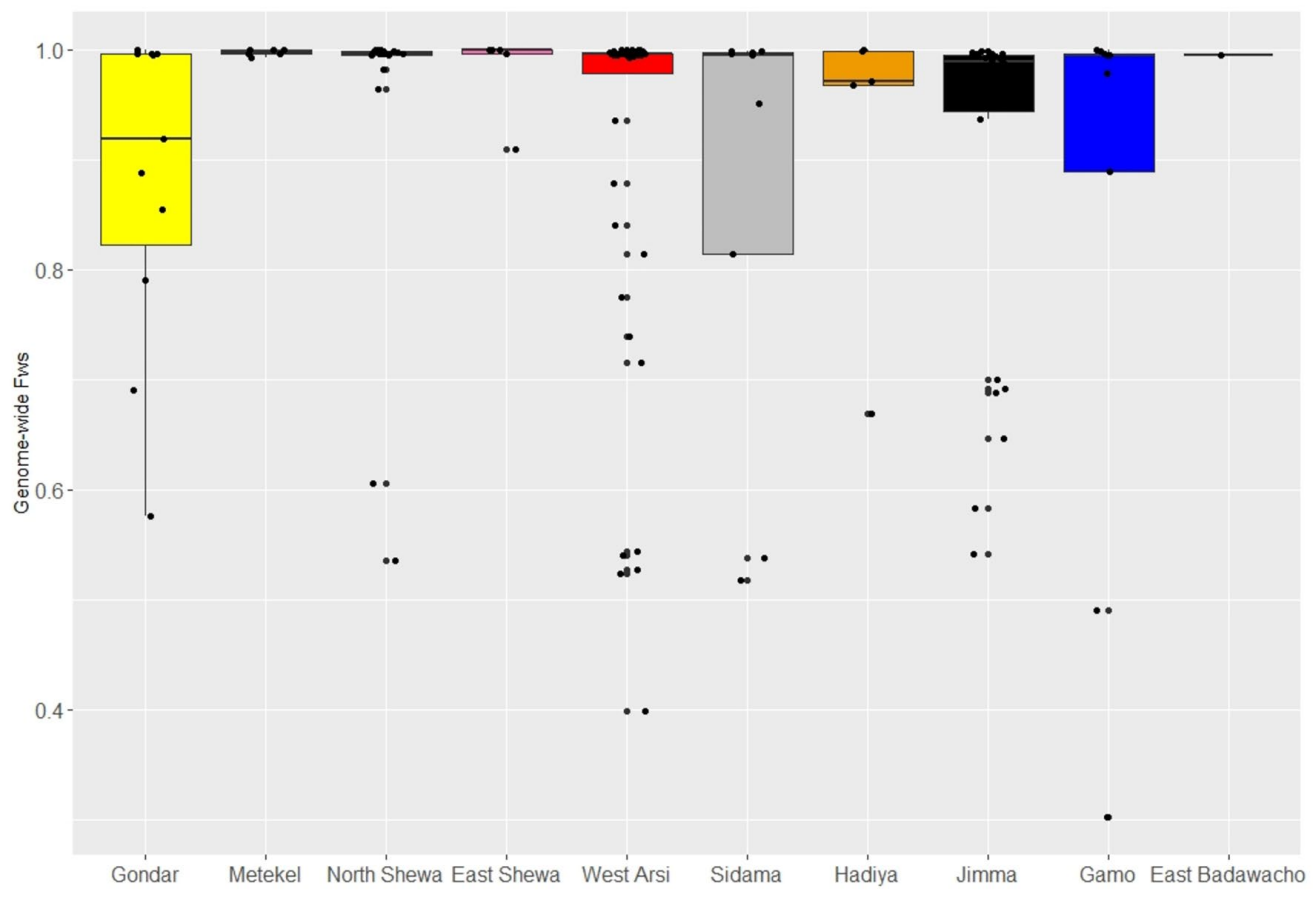
Within-sample infection complexity by district-level administration zone. Infection complexity was determined using the within-sample* F* statistic (*F*_WS_). The box and whiskers reflect the median, upper and lower quartile and 95% Confidence Intervals. The plot is based on data generated using *n* = 137 independent infections. Shewa Robit is a health centre in North Shewa district.

Within-sample infection complexity was assessed using the *F*_WS_ score, which ranges from 0 to 1, with increasing values reflecting increasing clonality. An *F*_WS_ threshold < 0.95 generally indicates polyclonal infection. Across Ethiopia, 25.5% (35/137) of infections had *F*_WS_ < 0.95. At the district level, the polyclonal infection prevalence ranged from 0 to 55%, with highest prevalence of polyclonality in Gondar (Fig. 2, Table 1). Median *F*_WS_ scores exceeded 0.95 in all districts aside from Gondar (median *F*_WS_ = 0.92, n = 11). When all nine districts were evaluated, there was no trend between malaria endemicity stratifications based on Annual Parasite Incidence (API) and the percentage of polyclonal infections. However, the small sample size at several sites may have constrained the accuracy of these estimates. Amongst the four sites with *n* ≥ 10 (North Shewa, West Arsi, Jimma and Gondar), a positive trend between endemicity and polyclonality was observed.

Plots of the non-reference allele frequency (NRAF) distributions across the genome were created for the 35 polyclonal infections, revealing a spectrum of within-host relationships (Supplementary Fig. 1). DEploid was used to phase the genomes of the clones making up ≥ 10% of an infection. The relatedness between the clones within each polyclonal infection was then evaluated using identity-by-descent (IBD) measures. In total 42.8% (15/35) of the isolates comprised at least one pair of clones with high relatedness (≥ 25% IBD) indicative of half-siblings or greater and likely reflecting co-infection (single mosquito inoculation, co-transmission) rather than superinfection (multiple inoculations) events. At the district-level, the prevalence of putative superinfections (defined as IBD < 25%) ranged from 0 to 100% (Table 1). Amongst the four sites with ≥ 10 isolates, there was no apparent trend between endemicity and superinfection prevalence.

### Subtle population structure between northern and southern regions of Ethiopia

**Figure 3:**
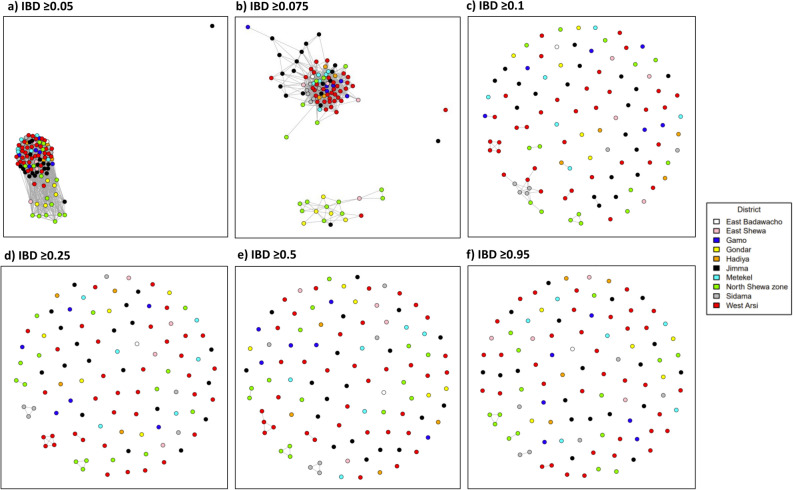
*P. vivax* connectivity in Ethiopia using measures of IBD. Panels (**a**–**f**) present cluster plots illustrating the relatedness between infections, as measured using identity by descent (IBD) measures of genetic distance, at thresholds ranging from 0.05 (minimum 5% genome shared) to 0.95 (minimum 95% genome shared, largely reflecting clones). At the 0.05 threshold, all infections aside from SGH-1-357 are connected to at least one other infection. At the 0.05 and 0.075 thresholds, the majority of isolates from Gondar and North Shewa form a distal cluster to the other districts. The isolates from Jimma also display relatively lower connectivity to the other districts. At the 0.1 threshold, geographic trends are less apparent, but multiple connections remain between infections within and between districts, particularly in North Shewa, West Arsi and Sidama. At the 0.25–0.95 thresholds (half-siblings and greater), connections are only observed within districts. The plots are based on data generated using n = 102 independent monoclonal infections.

IBD was used to capture patterns of relatedness between infections, both within and between districts. The median IBD between infections ranged from 5 to 10% across the districts, with the highest levels observed in Sidama (Table 1, Fig. 3). Connectivity plots illustrating relatedness at a spectrum of IBD thresholds (5, 7.5, 10, 25, 50 and 95%) revealed that, apart from one isolate from Jimma (SGH-1-357), all infections shared at least 5% of their genome with one other infection from across the Ethiopian data set (Fig. 3a). At the 7.5% IBD threshold, many of the infections from the northern districts of Gondar and North Shewa formed a distinct cluster from the other districts, and the isolates from Jimma revealed relatively lower connectivity relative to the other infections in the southern districts cluster (Fig. 3b). At thresholds above 10% IBD, the geographic trend across districts was less marked, and only small clusters of infection remained (Fig. 3c–f). As summarised in Supplementary Table 2, the levels of parasite connectivity between the districts in the northern and southern regions did not appear to be fully explained by geographic distance.

Neighbour-joining and ADMIXTURE analyses, which use distance measures based on Identity by State (IBS), confirmed subtle patterns of clustering amongst the isolates from Gondar and North Shewa, and Jimma, relative to the other districts (Supplementary Fig. 2a–c). The lowest CV error with ADMIXTURE analysis was at *K* = 5 (0.034), but there was a limited difference in the absolute number between *K* = 3 and *K* = 7 (0.034–0.035) (Supplementary Fig. 2d). At *K* = 3, aside from Gondar, the majority of isolates in each district exhibited predominant ancestry to the K1 sub-population. In Gondar, all isolates exhibited predominant ancestry to the K2 sub-population. Approximately 29.4% (5/17) of the isolates from North Shewa also exhibited predominant (> 80%) ancestry to K2. The K3 sub-population had the greatest representation in North Shewa, where 29.4% (5/17) of infections had predominant ancestry to this group. A large proportion (17/26, 65.4%) of the infections from Jimma displayed less than 80% ancestry to any given sub-population, rather, showing more mixed ancestry to the K1 and K2 sub-populations.

### Heterogeneity in prevalence of variants implicated in chloroquine, antifolate and mefloquine resistance

The prevalence of several variants that have previously been associated with clinical or ex vivo antimalarial drug resistance^28^, was determined for each of the nine districts with ≥ 4 isolates (Table 2). The most widely characterised candidate is the multidrug resistance 1 (*pvmdr1*) Y976F variant^29,30^, a minor modulator of chloroquine (CQ) resistance, had prevalence > 25% in all districts, and > 50% in North Shewa (6/12, 50.0%), West Arsi (24/36, 66.6%) and Gamo (3/6, 50.0%). However, the F1076L variant, which has also been implicated in CQ resistance^31^ was fixed at 100% frequency in all nine districts. None of the isolates had the *pvmdr1* copy number amplification associated with mefloquine resistance. A range of mutations in the dihydrofolate reductase (*pvdhfr*) and dihydropteroate synthase (*pvdhps*) genes have been associated with antifolate resistance^32–35^. The most common *pvdhfr* variants observed in Ethiopia were the S58R and S117N mutations, with double mutants present from 50 to 100% of isolates from across the districts. Neither the triple nor the quadruple *pvdhfr* mutants were observed in any district. At the *pvdhps* locus, the prevalence of A383G mutations was highly variable between sites, ranging from 0% in Metekel and Sidama to > 67% in East Shewa and Gondar, but no clear geographic trend was observed. No *pvdhps* A553G mutations were observed in any district.

**Table 2:** Prevalence of orthologous drug resistance markers in each district.

Gene	Chr	Position	Mutation	Drug	Frequency, % (no./No.)								
					Gondar	Metekel	North Shewa	East Shewa	West Arsi	Sidama	Hadiya	Jimma	Gamo
*pvmdr1*	10	479,908	F1076L	CQ	**100 (5/5)**	**100 (6/6)**	**100 (15/15)**	**100 (4/4)**	**100 (36/36)**	**100 (6/6)**	**100 (4/4)**	**100 (19/19)**	**100 (6/6)**
(PVP01_1010900)	10	480,207	Y976F	CQ, AQ + SP	*40 (2/5)*	*33 (2/6)*	**50 (6/12)**	*25 (1/4)*	**67 (24/36)**	*40 (2/5)*	*25 (1/4)*	*32 (6/19)*	**50 (3/6)**
	10	Copy number variant	≥ 2 copies	MQ	0 (0/5)	0 (0/2)	0 (0/13)	0 (0/2)	0 (0/30)	0 (0/4)	0 (0/1)	0 (0/17)	0 (0/4)
*pvdhfr-ts*	5	1,077,530; 1,077,532	F57L/I	Antifolate, AQ + SP	0 (0/5)	0 (0/6)	0 (0/15)	0 (0/4)	0 (0/36)	0 (0/6)	0 (0/4)	0 (0/19)	0 (0/6)
(PVP01_0526600)	5	1,077,533; 1,077,534; 1,077,535	S58R	Antifolate, AQ + SP	**100 (5/5)**	**100 (6/6)**	**100 (15/15)**	**75 (3/4)**	**75 (27/36)**	**100 (6/6)**	**50 (2/4)**	**89 (17/19)**	**100 (6/6)**
	5	1,077,543	T61M	Antifolate, AQ + SP	0 (0/5)	0 (0/6)	0 (0/15)	0 (0/4)	0 (0/36)	0 (0/6)	0 (0/4)	0 (0/19)	0 (0/6)
	5	1,077,711	S117N/T	Antifolate, AQ + SP	**100 (5/5)**	**100 (6/6)**	**100 (15/15)**	**100 (4/4)**	**92 (33/36)**	**100 (6/6)**	**100 (4/4)**	**95 (18/19)**	**100 (6/6)**
			Single mutant	Antifolate, AQ + SP	0 (0/5)	0 (0/6)	0 (0/15)	*25 (1/4)*	*17 (6/36)*	0 (0/6)	50 (2/4)	5 (1/19)	0 (0/6)
			Double mutant	Antifolate, AQ + SP	**100 (5/5)**	**100 (6/6)**	**100 (15/15)**	**75 (3/4)**	**75 (27/36)**	**100 (6/6)**	**50 (2/4)**	**89 (17/19)**	**100 (6/6)**
			Triple mutant	Antifolate, AQ + SP	0 (0/5)	0 (0/6)	0 (0/15)	0 (0/4)	0 (0/36)	0 (0/6)	0 (0/4)	0 (0/19)	0 (0/6)
*pvdhps*			Quadruple mutant	Antifolate, AQ + SP	0 (0/5)	0 (0/6)	0 (0/15)	0 (0/4)	0 (0/36)	0 (0/6)	0 (0/4)	0 (0/19)	0 (0/6)
(PVP01_1429500)	14	1,270,401	A553G	Antifolate	0 (0/5)	0 (0/6)	0 (0/15)	0 (0/4)	0 (0/36)	0 (0/6)	0 (0/4)	0 (0/19)	0 (0/6)
	14	1,270,911	A383G	Antifolate	**80 (4/5)**	0 (0/6)	*40 (6/15)*	**67 (2/3)**	6 (2/36)	0 (0/6)	*25 (1/4)*	*32 (6/19)*	*17 (1/6)*

Variants with frequency ≥ 10% but < 50% are highlighted in italics, and ≥ 50% in bold. Mutation prevalence was calculated with homozygous calls only. *AQ* amodiaquine, *Chr* chromosome, *CQ* chloroquine, *MQ* mefloquine, *SP* sulfadoxine-pyrimethamine.

### Substantial difference in frequency of a non-synonymous *pvaat1* variant between Ethiopia relative to Thailand and Indonesia

**Figure 4:**
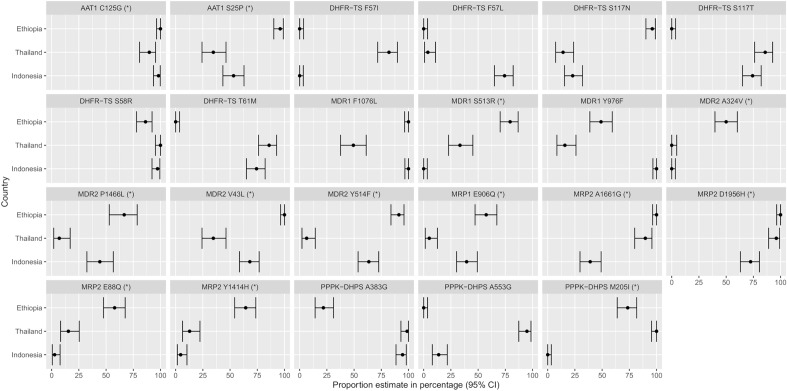
Amino acid frequencies in Ethiopia, Thailand and Indonesia at selected *P. vivax* drug resistance candidates. Each panel presents proportions and corresponding 95% confidence intervals (CIs) for the given amino acid changes. Data are provided on variants that have either (i) previously been associated with drug resistance (association with clinical phenotype) or (ii) are non-synonymous variants in orthologues of *P. falciparum* drug resistance-associated genes and exhibit substantial differences (non-overlapping CIs) in proportion between Ethiopia and Thailand or Indonesia; the latter class of variants are denoted with (*). For variants that have previously been associated with clinical or ex vivo resistance, frequencies reflect the drug-resistant amino acid, whilst frequencies for the other variants are relative to the reference strain (PvP01).

The molecular basis of antimalarial drug resistance is better understood in *P. falciparum* than *P. vivax*^16^. We therefore explored the prevalence of other non-synonymous variants in *pvmdr1*, *pvdhps*, *pvdhfr* and orthologues of several other genes implicated in *P. falciparum* resistance; *pvaat1* (amino acid transporter), *pvcrt-o* (chloroquine resistance transporter), *plasmepsin IV*, *pvmrp1* and *pvmrp2* (multidrug resistance-associated proteins 1 and 2) and *pvmdr2* (multidrug resistance protein 2). A summary is provided for Ethiopia and the comparator populations, Thailand and Indonesia, in Supplementary Data 2. Twenty-three non-synonymous variants displayed large differences in frequency, with non-overlapping confidence intervals between Ethiopia and Thailand or Indonesia (Fig. 4). The *pvaat1* S25P, three *pvdhfr* (S117N, S117T, T61M), *pvmdr1* S513R, four *pvmdr2* variants (A324V, P1466L, V43L and Y514F), *pvmrp1* E906Q, two *pvmrp2* (E88Q and Y1414H) and one *pvdhps* (A383G) variant displayed consistently different prevalence (> 20% higher or lower) between Ethiopia and the Asian populations, potentially reflecting Ethiopian-specific adaptations.

### Extended haplotype homozygosity reveals evidence of selection in the vicinity of *pvcrt-o* in Ethiopia

**Figure 5:**
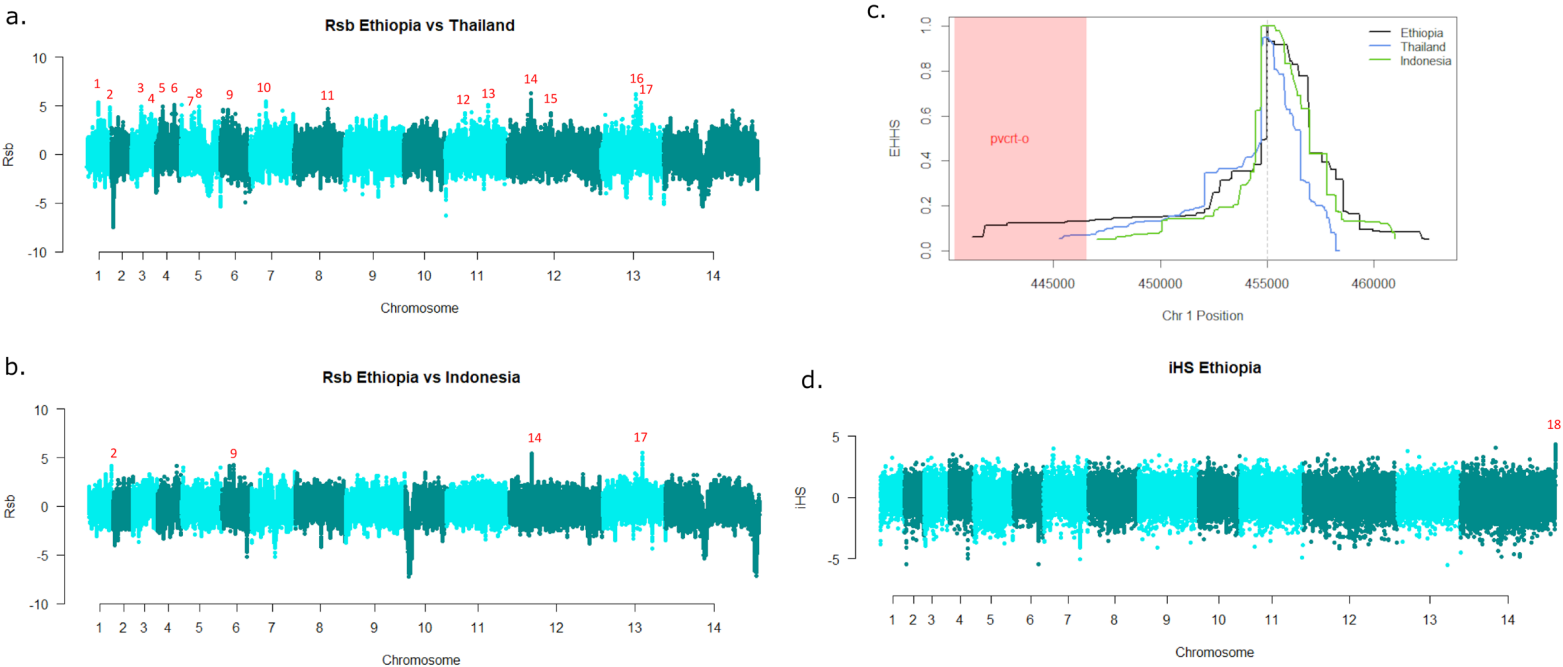
Patterns of extended haplotype homozygosity in Ethiopia. Panels (**a**) and (**b**) present Manhattan plots of the *Rsb*-based cross-population extended haplotype homozygosity (EHH) illustrating regions of divergent selection between Ethiopia and Thailand and Indonesia. Putative signals are numbered. Briefly, putative drivers include; chloroquine resistance transporter (*pvcrt*, PvP01_0109300) and prodrug activation and resistance esterase (PvP01_011010) at signal 1, lysine specific histone demethylase (PVP01_0118300) at signal 2, peptide chain release factor 2 (PvP01_0309000) at signal 3, phosphoinositide-binding protein PX1 (PvP01_0316400) at signal 4, acyl-CoA-synthetase (PvP01_0409900) at signal 5, merozoite surface protein (MSP) 4 and 5 (PVP01_0418300, PVP01_0418400) at signal 6, zinc finger protein (PvP01_0517200) at signal 7, serine/threonine protein phosphatase (PvP01_0603400) at signal 8, *pvkelch* protein K10 (PvP01_0607800) at signal 9, a tRNA (PVP01_0711200) at signal 10, PvP01_0824800 and PvP01_1115800 (unknown functions) at signals 11 and 12, oligomeric golgi complex subunit 4 (PvP01_1133300) at signal 13, an MSP7-like gene cluster at signal 14, intergenic region at signal 15, actin related protein ARP4 (PvP01_1326200) at signal 16 and liver-specific protein (PvP01_1330800) at signal 17. Panel (**c**) illustrates the comparative decay in EHH in the *pvcrt-o* region of signal 1. The dashed vertical grey line indicates the position at which the peak *Rsb* score was observed. The pink shaded region denotes the *pvcrt-o* region. While the peak *Rsb* is not located within *pvcrt-o*, haplotype homozygosity appears to decay less rapidly upstream (i.e., in the direction of *pvcrt-o*) than downstream of the signal peak. Ethiopia retains moderate haplotype homozygosity (EHHS > 0.1) within the *pvcrt-o* region. Panel (**d**) presents a Manhattan plot of the integrated haplotype score (*iHS*) illustrating regions under recent directional selection in Ethiopia alone. Only a single peak was detected in an intergenic region at signal 18. The data was derived from analyses on independent samples from Ethiopia (n = 102), Indonesia (n = 110) and Thailand (n = 87).

Analysis of other gene regions using measures of extended haplotype homozygosity, including the *Rsb* and *iHS* measures, provided evidence of recent directional selection. A previous Ethiopian study (*n* = 17 monoclonal samples) identified a weak signal of selection proximal to *pvcrt-o* in comparisons against Thailand but not Indonesia^25^; in the current analysis we focused comparisons of isolates from the same countries using the *Rsb* metric. Seventeen signals of directional selection were observed in Ethiopia relative to Thailand and four of these were also observed relative to Indonesia (Fig. 5a,b, Supplementary Table 3). Several drug resistance candidates were present within or proximal to these regions of selection, including a signal proximal to *pvcrt* with extended haplotypes in Ethiopia relative to Thailand (signal), confirming our previous findings (Fig. 5c)^25^. The signal 1 region includes another putative driver of drug resistance, a prodrug activation and resistance esterase (PvP01_011010). A signal in a putative driver for artemisinin resistance, the *pvkelch* 10 gene (PvP01_0607800, signal 9), that has previously been detected in Afghanistan, was exhibited in extended haplotypes present in Ethiopia relative to Indonesia^36^. A third drug-related signal of selection (signal 5) was observed in an orthologue of a new antimalarial target, acyl-CoA-synthetase (PvP01_0409900), with extended haplotypes in Ethiopia relative to Thailand^37,38^. Other signals of selection encompassed genes with a range of putative functions (detailed in Supplementary Table 3). Amongst these, signals 6 and 14 had peak *Rsb* scores in immune-related genes. Signal 6, is located in a region including a cluster of serine-repeat antigens (SERA), and merozoite surface protein (MSP) 4 and 5 (PVP01_0418300, PVP01_0418400), which are potential vaccine candidates^36,39–42^. The peak at signal 14 is in a gene with unknown function, but the region is downstream from a large MSP7-like gene cluster.

The integrated haplotype score (*iHS*) was also used to identify regions under apparent directional selection in Ethiopia as evidenced by relatively extended haplotypes flanking the alternate alleles at a given SNP. Only one major signal of directional selection (signal 18) was observed in Ethiopia, in a 3.5 kb region on chromosome 14 comprising 2 genes (Fig. 5d, Supplementary Table 3). The peak signal was in an intergenic region between two genes encoding *Plasmodium* exported proteins, PvP01_1470400 and PvP01_1470500.

### High frequency of duffy binding protein 1 copy number amplification

**Figure 6:**
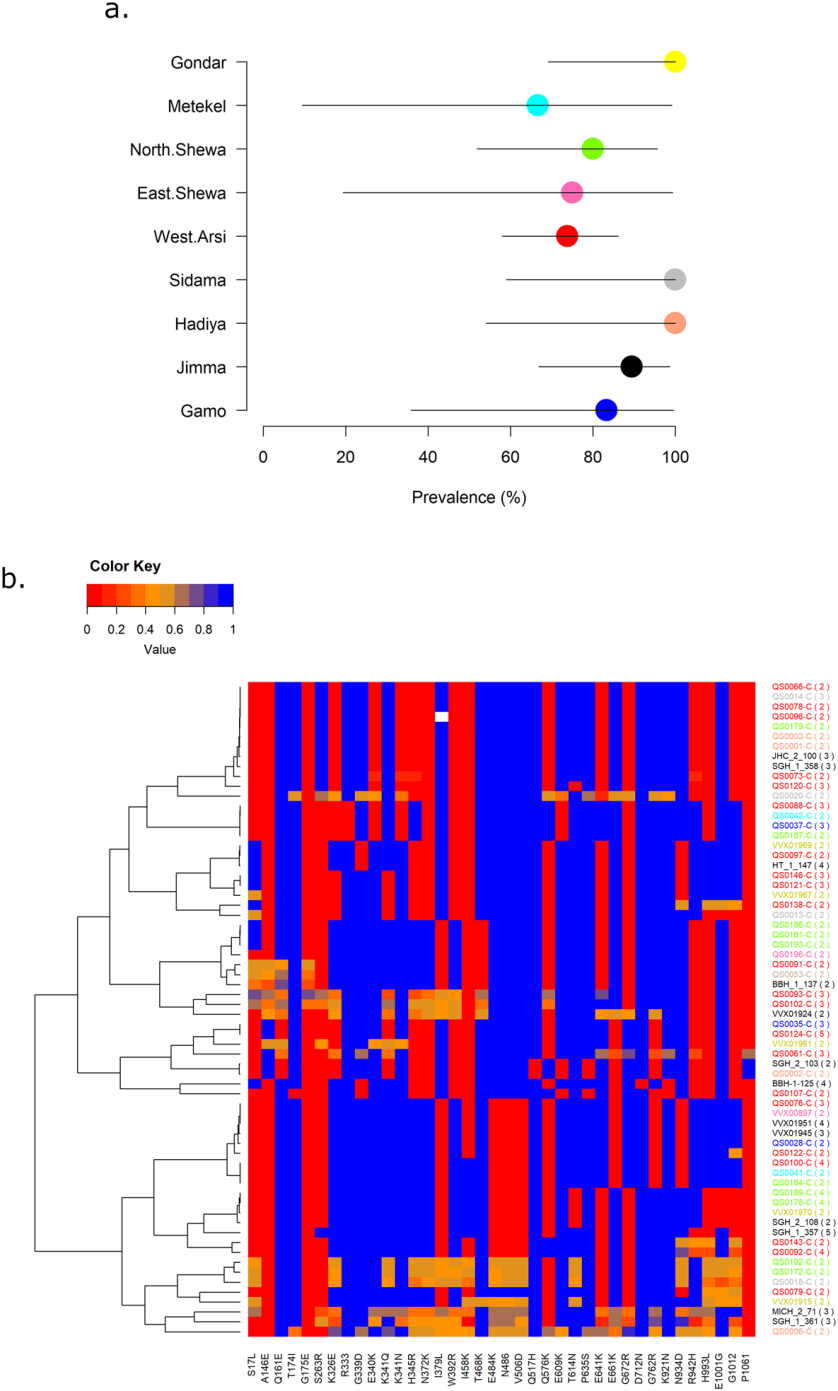
Prevalence and diversity of duffy binding protein 1 copy number amplifications in Ethiopia. Panel (**a**) illustrates the prevalence of the *pvdbp1* copy number amplification by district. The error bars reflect Clopper-Pearson confidence intervals. A high prevalence of *pvdbp1* copy number amplification (> 60%) was observed in all districts assessed. However, as illustrated by the standard error bars, sample size was constrained in districts such as Metekel and East Shewa. Panel (**b**) presents a heatmap illustrating the divergence between *pvdbp1* copies in monoclonal infections with copy number amplifications. Rows indicate samples and columns indicate amino acid changes conferred by underlying SNPs. Genotypes are shown as reference allele frequencies on a scale of 0 in red (homozygote alternative allele) to 1 in blue (homozygous reference allele). As the infections are monoclonal, heterozygotes (trending towards orange) are indicative of sequence divergence between the *pvdbp1* copies. Infections are clustered by genetic relatedness in the given sequence region. Sample identifiers (row labels) are colour-coded by district (as in panel a), and the number of *pvdbp1* copies is indicated in parentheses. Majority of samples have few heterozygotes (mostly red or blue genotypes) but several samples deriving from a range of districts have extensive divergence between *pvdbp1* copies (many orange genotypes). Panel (**a**) was generated using data on 102 independent samples, and panel (**b**) was generated using data on 102 independent, monoclonal samples with *pvdbp1* amplification.

The most prevalent copy number (CN) variant observed in Ethiopia was present in the *P. vivax* duffy binding protein 1 (*pvdbp1*), detected in 67.8% (93/112) of the analysable samples. Most *pvdbp1* CN amplifications harboured the previously described Cambodian breakpoint (88.2%, 82/93), with the remaining samples (11.8%, 11/93) having the Malagasy breakpoint (Supplementary Data 3)^43^. The number of *pvdbp1* copies in the amplifications ranged from 2 to 5 copies. Although some variation was observed between districts, all had a prevalence of *pvdbp1* CN amplification > 60% (Fig. 6a) with no apparent geographic trends.

The monoclonal isolates with *pvdbp1* CN amplification (*n* = 67) were further investigated to evaluate the additional genetic variation created by the extra gene copies. Amongst the 67 monoclonal samples, a mean of 3.4 heterozygous SNPs was observed in the CN region, indicative of modest sequence differences between the copies (Fig. 6b). However, the range of heterozygous SNPs ranged up to a maximum of 23 SNPs, highlighting the potential for extensive diversity between the *pvdbp1* copies.

## Discussion

Our large genomic study includes geographically widespread *P. vivax* Ethiopian isolates. It reveals high diversity in all geographic regions assessed, complex patterns of parasite connectivity between districts, and strong evidence of selection in a region proximal to the *pvcrt-o* locus. We confirm the high prevalence of the *pvdbp1* CN amplification in all geographic regions assessed. Herein we describe the epidemiological processes that may be shaping the observed population diversity and structure, and present hypotheses for the observed signals of selection.

Ethiopia has reported a steady rise of *P. vivax* infections from 2019 to 2021, and reported data systematically underestimates the true burden of infection as it does not account for the hidden reservoir of asymptomatic infections^1,3^. Current approaches to monitor *P. vivax* epidemiology using methods such as microscopy and rapid diagnostic tests (RDTs) are limited in detecting sub-microscopic and asymptomatic infections^44^. The dormant liver stage (hypnozoites) and splenic reservoirs further complicate efforts to infer the true toll of *P. vivax* infection in Ethiopia. Population genetic metrics can provide complimentary insights into parasite transmission, with *P. falciparum* studies revealing a positive correlation between transmission intensity and prevalence of polyclonal infections^45^. The situation in *P. vivax* is more complex, attributable in part to the hypnozoite reservoir^17,21^. In our study, 26% of infections were polyclonal, similar to our previous Ethiopian estimate of ~ 30% polyclonal infections^25^. These frequencies are lower than that observed in areas of intense *P. vivax* transmission such as Papua Indonesia (48%), but higher than in pre-elimination settings such as Malaysia (16%) or Panama (0%)^21,24,45^. A population genomic study conducted in northern Ethiopia (across Amhara, Gambella and Tigray) in 2017–18 detected a slightly lower level of polyclonal *P. falciparum* infections (~ 18%) potentially reflecting a contribution of the liver-stage reservoir to *P. vivax* transmission, but this needs to be investigated in co-endemic populations and with consideration of seasonal changes^46^. Our data also revealed marked polyclonal heterogeneity at the district level (0–55%), consistent with Ethiopia’s marked variation in malaria endemicity^25^. Although the sample size was limited in several districts, in the four districts with ≥ 10 isolates, there was a positive correlation between the API-based malaria endemicity (defined by the Ethiopian National Malaria Elimination Program (NMEP) and the frequency of polyclonal infections. In these four districts, polyclonality ranged from 12% in North Shewa (a pre-elimination settings), to 55% in Gondar (~ 400 km from North Shewa), more comparable to the intense transmission in Papua. Our findings concur with a microsatellite-based genotyping study, which revealed heterogeneity in *P. vivax* infection diversity between relatively proximal districts in southern Ethiopia^17^. Ecological, climatic and demographic factors are likely to impact malaria transmission in this region, but the limited sample size in several districts constrained our ability to conduct in-depth investigation of these factors. Further studies are needed with high-throughput genotyping across dense sample sets.

Our analysis of the relatedness (IBD) between the clones within polyclonal infections enabled further insights into *P. vivax* transmission, specifically concerning co-transmission (single mosquito inoculation carrying mixtures of parasite genomes) and superinfection (multiple mosquito inoculations)^47^. In blood meals with mixtures of parasite genomes, the obligate meiotic stage generates recombinant sporozoites that share parents, hence clones with evidence of recent IBD are more likely to have derived from the same mosquito inoculation than from different inoculations. Owing to the liver-stage reservoir, mixtures of unrelated *P. vivax* genomes can arise from infectious bites in close succession, or a recent inoculum combined with a reactivated hypnozoite from an older bite; both cases involve multiple inoculums. In concordance with our previous study in Ethiopia, almost half of all polyclonal infections carried at least two highly related clones (siblings or half-siblings, sharing ≥ 25% genomic IDB), suggestive of co-transmission events, with the remaining isolates presumably reflecting superinfections^25^. These findings infer a high proportion of both co-infection and superinfection events in Ethiopia. There was broad heterogeneity in the prevalence of putative superinfections between districts (0–100%). However, sample size was modest in several sites and our definition of superinfection (within-host IBD < 25%) may be imperfect and needs further exploration. Comparative evaluation of the IBD patterns in polyclonal infections in co-endemic *P. vivax* and *P. falciparum* populations will provide useful insights into the contribution of untreated hypnozoites to rates of *P. vivax* co-infection and superinfection, informing on transmission reduction priorities. It will also be important to explore within-host IBD patterns in sub-patent and asymptomatic infections, which may exhibit different dynamics than the symptomatic, patent reservoir^48^.

Information on the key drivers and barriers of infection spread between communities is critical for the national malaria elimination plan to decide how and where to prioritise interventions for maximum transmission reduction. The wide heterogeneity in the composition and abundance of Anopheline vector species, landscape features, agricultural practices and ethnic/cultural distributions complicate prediction of the main reservoirs and routes of parasite spread within and between communities of Ethiopia. Hence parasite genetic data has great potential to provide information on parasite connectivity between communities^49–51^. Previous studies using microsatellite data to measure IBS between *P. vivax* infections from different communities observed that geographic distance was not a major determinant of *P. vivax* infection spread in Ethiopia^17,52^. IBD has greater potential than IBS to capture connectivity between parasites in highly recombining species such as *Plasmodium* spp.^53,54^. Using both IBS and IBD measures on our genomic dataset, we confirmed that geographic distance was not a major driver of *P. vivax* connectivity in Ethiopia. Whilst isolates from the northern districts of Gondar and Shewa Robit could be differentiated from the southern districts, the northern district of Metekel had limited connectivity with neighbouring Gondar, instead exhibiting greater connectivity with the southern districts. This area is the epicentre of the Grand Ethiopian Renaissance Dam (GERD), the biggest hydroelectric dam in Africa, which has been under construction since 2011, employing between 8500–12,000 people from across Ethiopia at its peak. The high connectivity between the *P. vivax* populations in Metekel and the southern districts may therefore reflect a large influx of migrant workers from these regions. Although Gondar and Shewa Robit are > 400 km apart, the high connectivity is consistent with historical cultural ties between these districts^55^. Another distinct pattern observed in our dataset was moderate differentiation between Jimma and neighbouring districts in the south of the country. The patterns of gene flow with Jimma may reflect dense forest in the region that could impede movement of people from nearby districts. Our results suggest that economic and cultural factors are likely to be important drivers of human mobility and associated *P. vivax* infection spread in Ethiopia. These factors can be difficult to predict but combining parasite genetics with other data on human mobility such as mobile phone data, may provide critical information on connectivity between communities, as has been demonstrated in *P. falciparum* studies^49,50^.

Defining antimalarial drug resistance in *P. vivax* is challenging^16,28^. Clinical efficacy of treatment regimens for *P. vivax* is confounded by recurrent infections which can arise from recrudescence, reinfection or relapse. Chloroquine (CQ) remains the mainstay of treatment for *P. vivax* in Ethiopia and most vivax endemic countries, although high grade CQ resistance (CQR) has been reported from Indonesia, Papua New Guinea and Malaysia. In most other endemic areas, there have also been sporadic reports of low grade CQR^56^. Although some therapeutic efficacy studies have documented declining CQ efficacy in Ethiopia^7–11,13,14,57^, others report sustained high efficacy^12,58–60^. The local *P. vivax* population has also been subject to selection pressure from artemether-lumefantrine and other antimalarials targeting the co-endemic *P. falciparum* population. Sulfadoxine-pyrimethamine (SP) and CQ were withdrawn from treatment guidelines for *P. falciparum* more than three decades ago owing to their poor efficacy^61^. However, SP is still recommended for intermittent preventive treatment in pregnancy (IPTp), infancy (IPTi) and childhood (IPTc) for all malaria species^62–64^. Our study revealed a high frequency of *pvdhfr* double mutants (58R/117N) in all districts evaluated (50–100%). Studies have shown that these *pvdhfr* double mutants can induce up to 460-fold increase in resistance to pyrimethamine^65^. In contrast to many Southeast Asian populations, no isolates with triple or quadruple *pvdhfr* mutations were observed in Ethiopia, suggesting lower selective pressure from SP^25^. The apparent lower selective pressure in Ethiopia may reflect a lower rate of drug implementation than in Thailand or Indonesia. At the *pvdhps* locus, the wildtype 553A variant predominated, but heterogeneity was observed between districts in the prevalence of the resistance-associated 383G variant. Further evaluation of the impact of the combined 58R/117N/553A/383G genotype on SP efficacy in IPTp and IPTi is required.

Our study also determined the prevalence of *P. vivax* multidrug resistance 1 (*pvmdr1*) polymorphisms. In keeping with the limited use of mefloquine in Ethiopia, there was no evidence of *pvmdr1* copy number amplification, which has been associated with mefloquine resistance^19^. Single nucleotide polymorphisms at *pvmdr1* 976 and 1076 have been implicated in CQR^29,31^. There was a high prevalence of the *pvmdr1* 976F mutation (25–67%), and *pvmdr1* 1076L was at fixation^6,16,29^. However, the significance of these mutations is uncertain as they may be only minor modulators of CQR. The major molecular determinant of CQR in *P. falciparum* is the chloroquine resistance transporter (*pfcrt*)^66,67^. Although several studies have proposed that the *pfcrt* orthologue (*pvcrt-o*) has a role in *P. vivax* CQR, this remains contentious^6,16^. In a previous genomic analyses, we reported extended haplotype homozygosity in a region proximal to *pvcrt-o* in Ethiopia relative to Thailand (where CQR prevalence was low) but not Papua Indonesia (where CQR prevalence was high); however, the study was constrained by small sample size with only 17 monoclonal Ethiopian isolates^25^. In the current study, we confirmed the signal in the *pvcrt-o* region using 102 monoclonal Ethiopian isolates. However, this signal region contains other gene candidates, including a prodrug activation and resistance esterase (PvP01_011010), which may also be potential drivers of the observed haplotype homozygosity. The epicentre of CQR in *P. vivax* is in Papua, Indonesia, and yet comparisons between Indonesia and Thailand found no evidence of extended haplotypes in the *pvcrt-o* region^24,25^. This difference could reflect variation in demographic or selective pressures, or potentially different mechanisms of CQR between populations. Further functional studies of *pvcrt-o* are needed to explore this further^68^.

A recent study has implicated the amino acid transporter 1 gene (*pfaat1*) in the evolution of chloroquine resistance in *P. falciparum*^27^. Gene editing demonstrates that *pfaat1* S258L potentiates CQ resistance at a fitness cost, while a common southeast Asian variant (*pfaat1* F313S) reduces CQ resistance while restoring fitness^27^. We found no evidence of extended haplotypes at *pvaat1* in Ethiopia (or Thailand or Indonesia). However, we did observe substantially higher frequency of a non-synonymous variant (*pvaat1* S25P) in Ethiopia (96%) than Thailand (35%) or Indonesia (53%), potentially reflecting local differences in CQ resistance evolution. Three-dimensional protein structure models of *pvaat1* will be helpful to discern the impact of *pvaat1* S25P on drug transportation in *P. vivax*.

Our study also highlighted selection of regions coding for other putative drivers of drug resistance. Most prominent of these was the *P. vivax* kelch 10 gene (PvP01_0607800), an orthologue of *P. falciparum kelch13*, which has been associated with artemisinin resistance^69,70^. However, the implications of this with regard for artemisinin resistance in *P. vivax* are unclear; clinical efficacy studies have shown that artemisinin-based combination therapies (ACTs) including artemether-lumefantrine (AL), retain potent efficacy against *P. falciparum* and *P. vivax* in Ethiopia^11^. A recent study revealed 8% prevalence of a *P. falciparum* candidate artemisinin partial resistance *kelch13* R622I mutation across three regions of Ethiopia^46^. Although the R622I mutant has not been validated as a determinant of artemisinin resistance, close monitoring of both *P. falciparum* and co-endemic *P. vivax* populations for early assigns of ACT resistance is a priority.

The low prevalence of *P. vivax* in most of sub-Saharan Africa has been attributed to the predominance of the human Duffy negative genotype, which prevents expression of the Duffy Antigen Receptor for Chemokine (DARC) in red blood cells (RBCs)^2,71,72^. For several decades, DARC was thought to be critical for *P. vivax* invasion of human RBCs, mediated by a parasite duffy binding protein encoded by *pvdbp1*^71^. However, there are increasing reports of *P. vivax* infection in DARC (Duffy) negative individuals in Ethiopia^73^. The rate of Duffy negativity ranges from 3 to 35% in Ethiopia, providing a setting in which Duffy heterozygous individuals could prime *P. vivax* adaptations enabling invasion of Duffy negative RBCs^52,73–75^. An alternative hypothesis is that other pathways may facilitate *P. vivax* to invade Duffy negative individuals^76^. There was a high prevalence of *pvdbp1* copy number amplifications (67.8% infections carrying two or more copies) in all districts assessed, with evidence of at least two independent origins, with some isolates exhibiting up to 5 gene copies; these findings suggest major adaptive functions across Ethiopia. It’s possible that *pvdbp1* amplification may enable low-affinity binding of *P. vivax* to Duffy negative RBCs via an alternative receptor to DARC^26^, or *pvdbp1* amplification may allow for mutations in the extra gene copies, which could enhance diversity and immune evasion^26^. Indeed, we observed evidence of moderate diversity between *pvdbp1* copies in several infections evaluated. In line with the immune evasion hypothesis, a study in Cambodia found evidence that *pvdbp1* amplification reducing humoral immunity against *P. vivax*^72^. The prevalence of *pvdbp1* amplification in Ethiopia is the highest reported globally^77^, and may reflect an adaptation supporting both RBC invasion and immune evasion mechanisms.

In summary, the genomic architecture of *P. vivax* in Ethiopia highlights adaptations of potential public health concern in an endemic setting with evidence of stable transmission and long-distance spread of infection. Our findings highlight the need for more dense and geographically widespread molecular data that can be generated in a timely manner to facilitate effective surveillance and response.

## Methods

### Samples and study sites

All genomic data used in the study was derived from the open-access malaria Genomic Epidemiology Network (MalariaGEN) *P. vivax* genome variation project (Pv4.0) dataset^77^. The Ethiopian *P. vivax* genomic data were derived from patient isolates collected in the framework of clinical surveys and cross-sectional studies conducted in nine districts across northern and southwestern Ethiopia between 2012 and 2016 (Fig. 1). Briefly, published *P. vivax* genomes were obtained from cross-sectional surveys undertaken in Arbaminch, Badowacho, Hawassa and Halaba between May and November 2013, and Jimma between September and November 2016^18,25^. Additional samples were collected from two frameworks: a previously described clinical survey of Tafenoquine efficacy in *P. vivax*-infected patients recruited from Gondar and Jimma in 2016^78^, and a cross-sectional survey conducted in West Arsi, East Shewa (Adama) and Shewa Robit (North Shewa) between February 2013 and October 2015^77^. Details on the publication status of the samples can be found in Supplementary Data 1. Based on diverse ecological risk factors that correspond to a range of transmission intensity and pattern, malaria transmission was stratified into five strata: high malaria endemicity, moderate malaria endemicity, low malaria endemicity, very low malaria endemicity and malaria-free areas^79^. Three of our study sites, Sidama, Gamo and Metekel represent areas of highly endemic malaria, with stable year-round transmission, supported by hot, humid, tropical climates. Hadiya, Jimma and West Arsi represent moderate malaria endemic areas with moderate, seasonal transmission. Shewa Robit, Gondar and East Shewa (Adama) are areas with overall very low malaria endemicity, but a short period of intense malaria transmission. In Ethiopia, the first line antimalarial for treating *P. vivax* infection is chloroquine whereas artemether-lumefantrine is used to treat monoclonal *P. falciparum* or mixed-species (*P. falciparum*, *P. vivax)* infections.

Comparative analyses were undertaken using MalariaGEN Pv4.0 data from Thailand and Papua Indonesia^25^. The Thai population represented a region with low-grade chloroquine resistance, while the Papua Indonesia population represented a region with high-grade chloroquine resistance^80,81^. The samples were collected from symptomatic patients attending outpatient clinics in Tak Province, Thailand (2006–2013) and Mimika district, Papua Indonesia (2011–2014). The frontline treatment for *P. vivax* infection at the time of the enrollments was chloroquine plus primaquine in Thailand and dihydroartemisinin-piperaquine plus primaquine in Indonesia.

### Isolates and sequence data used for analysis.

The study used genome-wide Single Nucleotide Polymorphism (SNP) and Copy Number Variant (CNV) data from the MalariaGEN Pv4.0 release^77^. All Pv4.0 data are derived from *P. vivax* patient samples that were sequenced using Illumina platforms with paired end reads. In brief, after exclusion of any human reads, *P. vivax* sequence data from MalariaGEN partners and selected published data sets were mapped to the *P. vivax* P01 v1 reference genome using bwa^82,83^. GATK Best Practices Workflows were used to call SNPs and minor insertions and deletions (indels)^84^. The resulting Variant Calling Format (VCF) file, describing ~ 4.5 million variants in 1895 *P. vivax* samples is publicly available on the MalariaGEN website (https://www.malariagen.net/data/open-dataset-plasmodium-vivax-v4.0). CNVs due to large tandem duplications (> 3 kbp) were genotyped using a two-stage process where breakpoints were first discovered at base pair resolution using a combination of read depth and split reads and then sample were genotyped at these discovered breakpoints using a combination of read depth and read pairs mapped in a tail-to-tail configuration. Details on these CNVs, including copy number amplifications of the *pvmdr1* and *pvdbp1* genes are also available within the open access Pv4.0 data release^77^.

From the initial Pv4.0 VCF, only independent (i.e., no recurrence pairs or duplicates) samples from Ethiopia (n = 159), Thailand (n = 129) and Indonesia (n = 191) were extracted. Sample and SNP filtering processes were performed to exclude low-quality variants and reduce genotyping failures (defined as positions with < 5 reads). For genome-wide population genomic analyses, a VCF was generated by restricting to high quality (VQSLOD > 0) biallelic SNPs with < 5% sample failure rate and samples with < 15% genotype failure at the given SNPs. The final population genomic analysis set (from Ethiopia (n = 137), Thailand (n = 104), and Indonesia (n = 111)) contained 448 samples and 410,900 SNPs. The list of Ethiopian isolates along with European Nucleotide Archive accession numbers is provided in Supplementary Table 1. For candidate drug resistance analysis, a second VCF was generated that contained all high quality (VQSLOD > = 0) variants (with no filtering by genotyping failure or biallelic status) within previously described drug resistance candidates, with no further sample filtering^28^.

### Data analysis

The within-sample *F* statistic (*Fws*) was used to evaluate within-host infection complexity^85,86^. *Fws* > 0.95 was utilised as a cutoff to represent monoclonal infections. Within-host infection complexity was further explored using *DEploid* software to deconvolve polyclonal infections^87,88^. Briefly, all Ethiopian samples were used to create a population level allele frequency (PLAF) file. The samples were then scanned with *DEploid* to identify samples with more than one clone with 10% or more proportions. To create a reference panel, only samples with *Fws* > 0.95 and only one clone with 10% or more proportions were included. Samples that were not included in the reference panel were deconvolved with *DEploid-BEST* (https://github.com/DEploid-dev/DEploid). Deconvolved haplotypes were only derived on clones making up a minimum 10% of the total infection composition as estimated by allele frequency. Using the deconvolved haplotype reconstructions, *hmmIBD* software was used to determine the identity by descent (IBD) between the clones within each infection using default parameters^89^. The genome-wide patterns of within-host diversity were also illustrated with Manhattan plots of the non-reference allele frequency (NRAF). All further analyses were restricted to the monoclonal isolates (*Fws* > 0.95).

Spatial patterns of infection relatedness within Ethiopia were assessed using IBD measures between infections, calculated using *hmmIBD*^89^*.* IBD thresholds ranging from 0.05 (minimum 5% genome shared) to 0.95 (minimum 95% genome shared) were illustrated using network plots created using the *R*-based *igraph* package (https://igraph.org). Parasite relatedness was also assessed with neighbour-joining (NJ) analysis, using the *R* Adegenet package to compute a pairwise distance matrix and iTOL software to plot a tree^90^. The distance matrix was also used to conduct principal coordinate analysis (PCoA) using the R-based *adegenet* package^91^. ADMIXTURE analysis was used to infer population structure, and the cross-validation error was used to determine the most likely number of subpopulations (*K*)^92^.

Signals of selection were explored using the *R*-based *rehh* software to calculate the integrated haplotype score (*iHS*) and the *Rsb* measure of cross-population extended haplotype homozygosity (XP-EHH). The Thai and Indonesian populations were used as comparator populations for the XP-EHH analysis. Using similar thresholds to previous studies, signals supported by ≥ 3 SNPs above a threshold –log10 (*P* value) > 4 within 50 kb of one another and with an overall SNP density < 10 kb per SNP are numbered^25,36^.

### Ethics

As detailed in the MalariaGEN Pv4.0 data release, all *P. vivax* patient isolates used in the study were collected with local ethics approval, and written informed consent was obtained from all participants or a legal guardian where participants were ≤ 18 years of age^77^. In addition to the Pv4.0 release, several of the Ethiopian genomes have been published elsewhere^25,78,93^. Ethical approval for patient sampling from the new study sites in East Shewa, West Arsi and Shewarobit was granted by the ethics boards of Aklilu Lemma Institute of Pathobiology, Addis Ababa University, Armauer Hansen Research Institute, the national research ethics, the London School of Hygiene and Tropical Medicine. All methods were performed in accordance with the guidelines and regulations of the listed ethics committees.

### Supplementary Information

Supplementary Information 1.

Supplementary Information 2.

Supplementary Information 3.

Supplementary Information 4.

## Data Availability

The raw sequencing data for the 137 high-quality Ethiopian *P. vivax* samples described in the study are all openly accessible in the European Nucleotide Archive, with accession codes detailed in Supplementary Data 1. As described in the methods, the *P. vivax* genotyping data used for the Ethiopian and global analyses all derives from the MalariaGEN Pv4.0 dataset and is accessible as a Variant Calling Format (VCF) file (describing ~ 4.5 million variants in 1895 worldwide *P. vivax* genomes) on the MalariaGEN website at https://www.malariagen.net/data/open-dataset-plasmodium-vivax-v4.0.
