# Supplementary material for: Genomic analysis of Plasmodium vivax describes patterns of connectivity and putative drivers of adaptation in Ethiopia
Source: Sci Rep. 2023 Nov 27;13:20788. doi: 10.1038/s41598-023-47889-w (PMC10682486; doi:10.1038/s41598-023-47889-w)
Supplement: Supplementary file 1 — Supplementary Information 1. [file 41598_2023_47889_MOESM1_ESM.pdf]

Supplementary Information for:
Genomic analysis of *Plasmodium vivax* describes patterns of connectivity and putative drivers of adaptation in Ethiopia

Alebachew Messele Kebede[1], Edwin Sutanto[2], Hidayat Trimarsanto[3,4], Ernest Diez Benavente[5], Mariana Barnes[3], Richard D Pearson[6], Sasha V Siegel[6], Berhanu Erko[1], Ashenafi Assefa[7,8], Sisay Getachew[9,10,11], Abraham Aseffa[9], Beyene Petros[10], Eugenia Lo[12], Rezika Mohammed[13], Daniel Yilma[14], Angela Rumaseb[3], Francois Nosten[15,16], Rintis Noviyanti[4], Julian C. Rayner[17], Dominic P Kwiatkowski[6]*, Ric N Price[3,16,18], Lemu Golassa[1], Sarah Auburn[3,16,18]**

1. Aklilu Lemma Institute of Pathobiology, Addis Ababa University, Addis Ababa, Ethiopia
2. Exeins Health Initiative, Jakarta, Indonesia
3. Menzies School of Health Research and Charles Darwin University, Darwin, Australia
4. Eijkman Institute for Molecular Biology, Jakarta, Indonesia
5. Laboratory of Experimental Cardiology, Department of Cardiology, University Medical Center Utrecht, Utrecht, the Netherlands
6. Wellcome Sanger Institute, Hinxton, UK
7. Ethiopian Public Health Institute, Addis Ababa, Ethiopia
8. School of Public Health, Addis Ababa University, Addis Ababa, Ethiopia
9. Armauer Hansen Research Unit (AHRI), Addis Ababa, Ethiopia
10. Addis Ababa University, Addis Ababa, Ethiopia
11. MilliporeSigma (Bioreliance), Rockville, USA
12. Department of Microbiology and Immunology, College of Medicine, Drexel University Philadelphia, USA
13. University of Gondar, Gondar, Ethiopia
14. Jimma University Clinical Trial Unit, Department of Internal Medicine, Jimma University, Jimma, Ethiopia
15. Shoklo Malaria Research Unit, Faculty of Tropical Medicine, Mahidol University, Mae Sot, Thailand
16. Centre for Tropical Medicine and Global Health, Nuffield Department of Medicine, University of Oxford, Oxford, UK
17. Cambridge Institute for Medical Research, University of Cambridge, Cambridge, UK
18. Mahidol-Oxford Tropical Medicine Research Unit, Mahidol University, Bangkok, Thailand

*Deceased 24th April 2023
**Corresponding author: A/Prof Sarah Auburn, Sarah.Auburn@Menzies.edu.au, Menzies School of Health Research, PO Box 41096, Casuarina, Darwin, NT 0811, Australia; Tel: (+61) 8 8946 8503

Contents
Supplementary Figure 1. 1
Supplementary Figure 2. 7
Supplementary Table 1. 8
Supplementary Table 2. 9
Supplementary Table 3. 10

1.

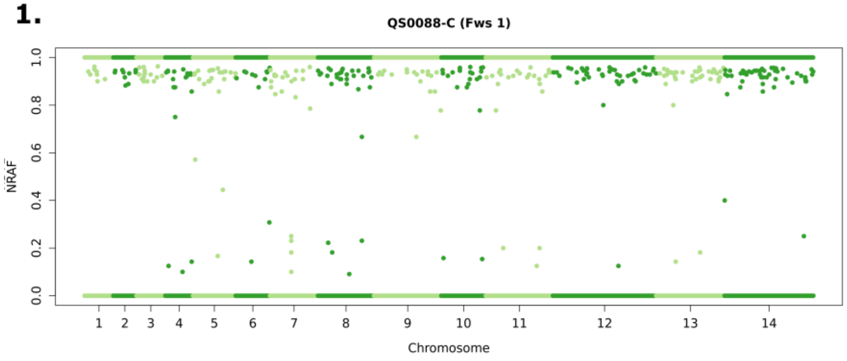
QS0088-C (Fws 1)
NRAF
0.0 0.2 0.4 0.6 0.8 1.0
1 2 3 4 5 6 7 8 9 10 11 12 13 14
Chromosome

2.

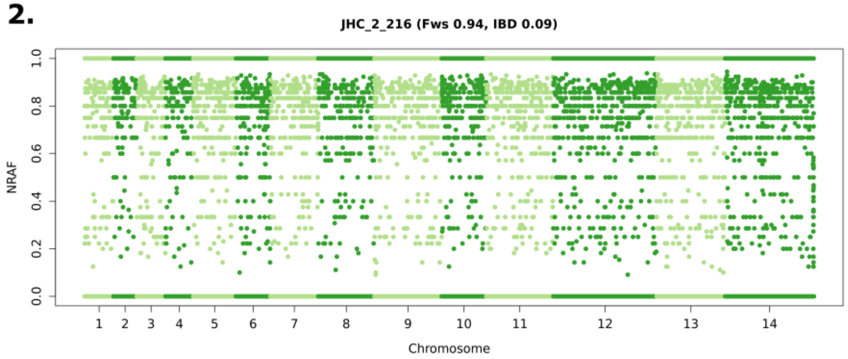
JHC_2_216 (Fws 0.94, IBD 0.09)
NRAF
0.0 0.2 0.4 0.6 0.8 1.0
1 2 3 4 5 6 7 8 9 10 11 12 13 14
Chromosome

3.

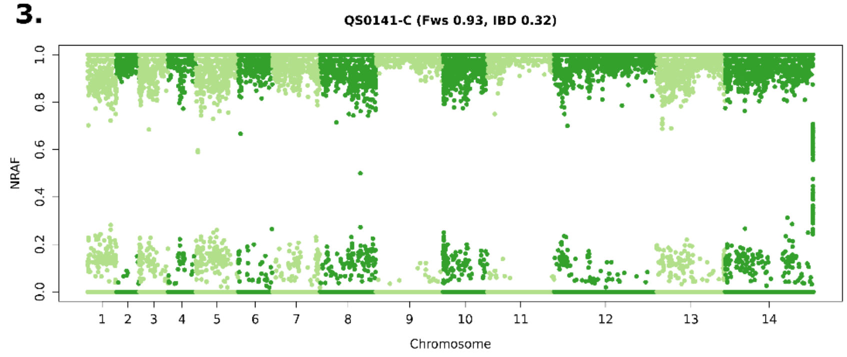
QS0141-C (Fws 0.93, IBD 0.32)
NRAF
0.0 0.2 0.4 0.6 0.8 1.0
1 2 3 4 5 6 7 8 9 10 11 12 13 14
Chromosome

4.

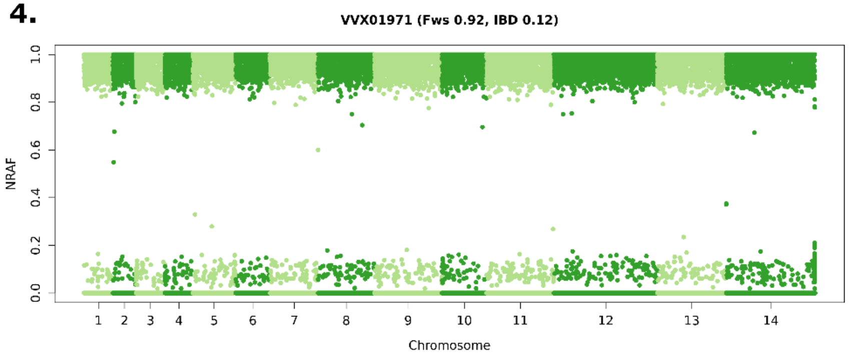
VVX01971 (Fws 0.92, IBD 0.12)
NRAF
0.0 0.2 0.4 0.6 0.8 1.0
1 2 3 4 5 6 7 8 9 10 11 12 13 14
Chromosome

5.

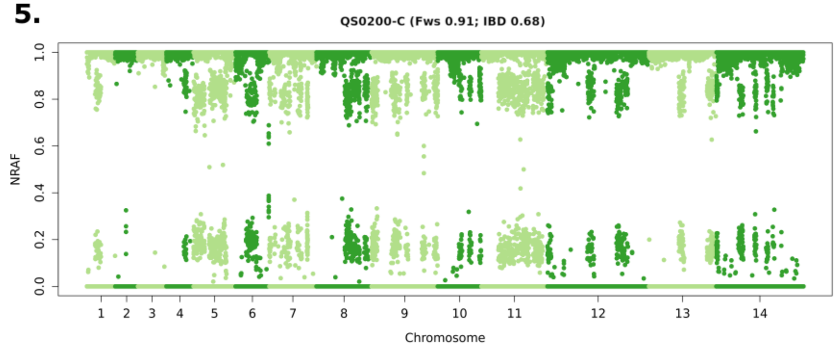
QS0200-C (Fws 0.91; IBD 0.68)
NRAF
0.0 0.2 0.4 0.6 0.8 1.0
1 2 3 4 5 6 7 8 9 10 11 12 13 14
Chromosome

6.

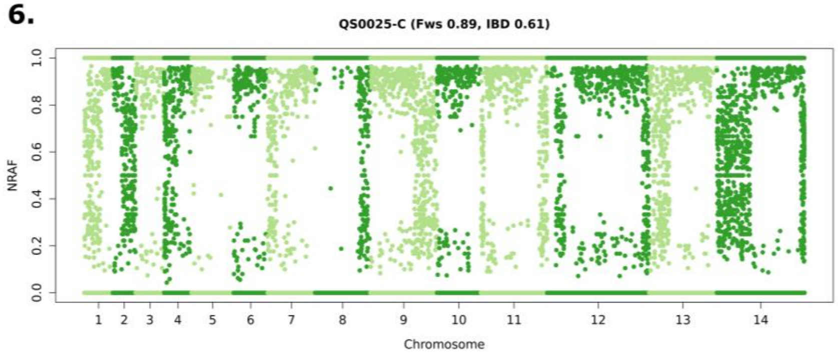
QS0025-C (Fws 0.89, IBD 0.61)
NRAF
0.0 0.2 0.4 0.6 0.8 1.0
1 2 3 4 5 6 7 8 9 10 11 12 13 14
Chromosome

7.

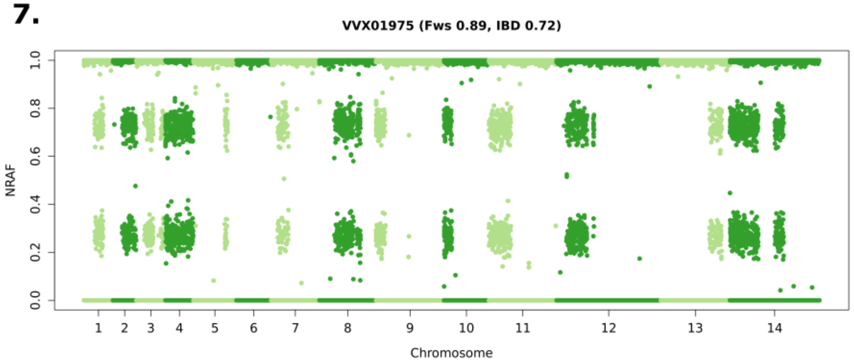
VVX01975 (Fws 0.89, IBD 0.72)
NRAF
0.0 0.2 0.4 0.6 0.8 1.0
1 2 3 4 5 6 7 8 9 10 11 12 13 14
Chromosome

8.

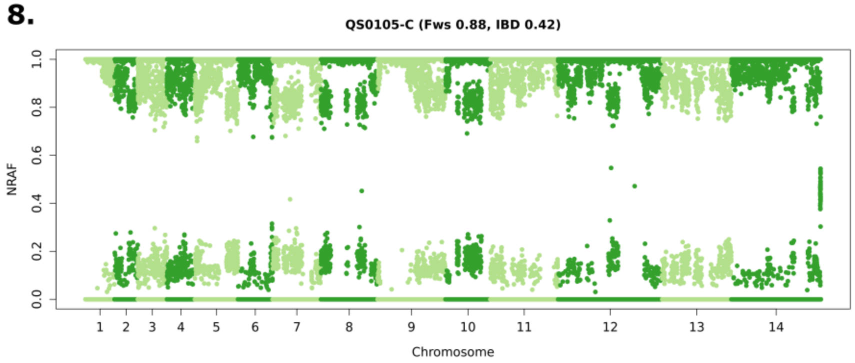
QS0105-C (Fws 0.88, IBD 0.42)
NRAF
0.0 0.2 0.4 0.6 0.8 1.0
1 2 3 4 5 6 7 8 9 10 11 12 13 14
Chromosome

9.

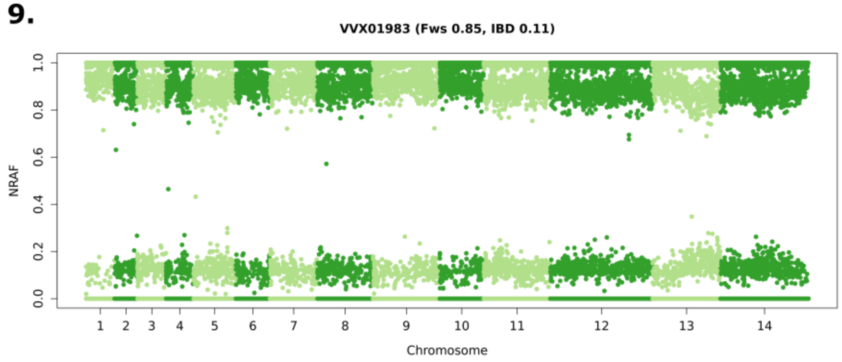
VVX01983 (Fws 0.85, IBD 0.11)
NRAF
0.0 0.2 0.4 0.6 0.8 1.0
1 2 3 4 5 6 7 8 9 10 11 12 13 14
Chromosome

10.

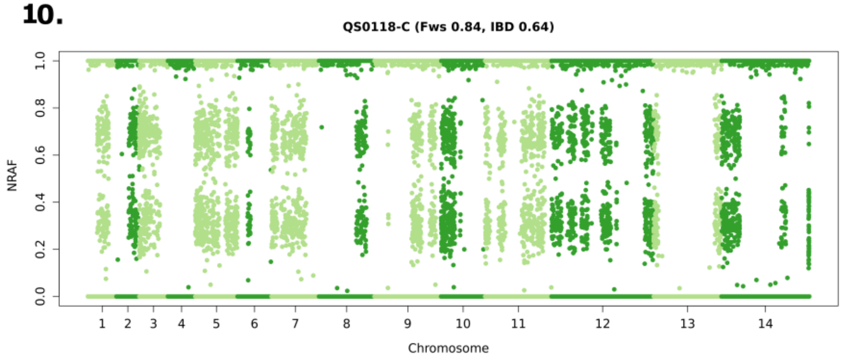
QS0118-C (Fws 0.84, IBD 0.64)
NRAF
0.0 0.2 0.4 0.6 0.8 1.0
1 2 3 4 5 6 7 8 9 10 11 12 13 14
Chromosome

11.

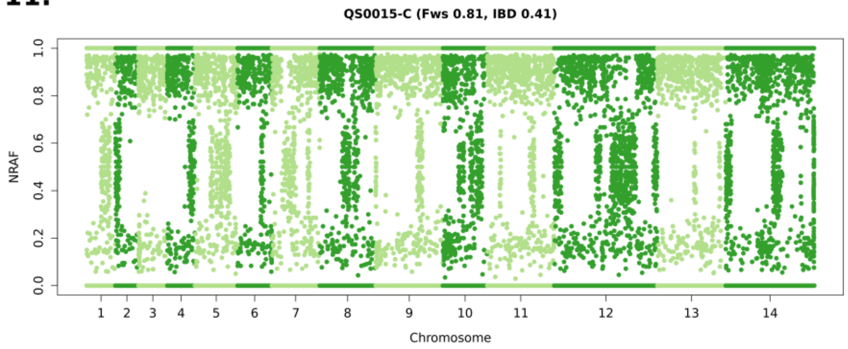
QS0015-C (Fws 0.81, IBD 0.41)
NRAF
0.0 0.2 0.4 0.6 0.8 1.0
1 2 3 4 5 6 7 8 9 10 11 12 13 14
Chromosome

12.

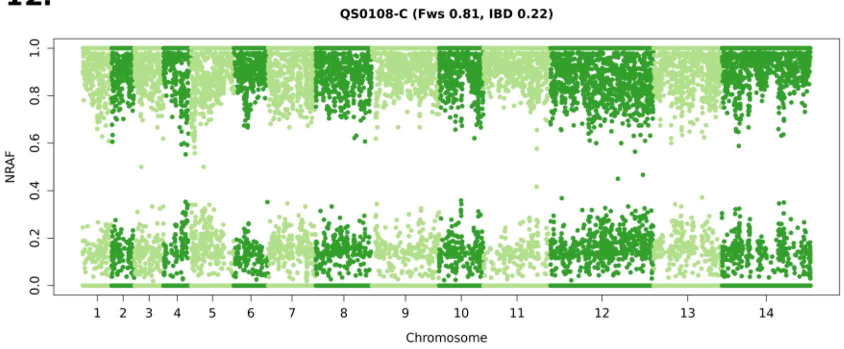
QS0108-C (Fws 0.81, IBD 0.22)
NRAF
0.0 0.2 0.4 0.6 0.8 1.0
1 2 3 4 5 6 7 8 9 10 11 12 13 14
Chromosome

13.

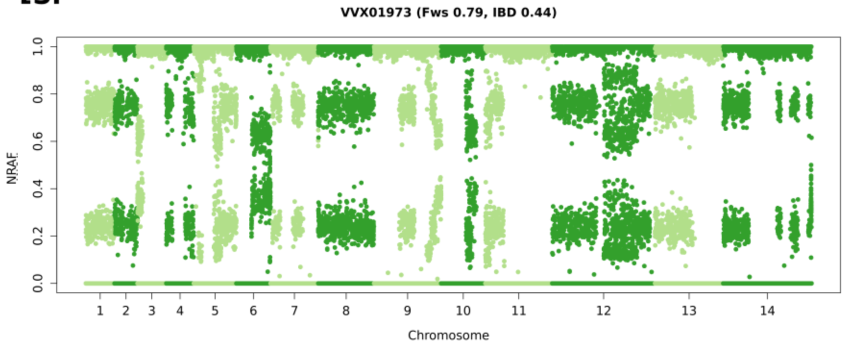

14.

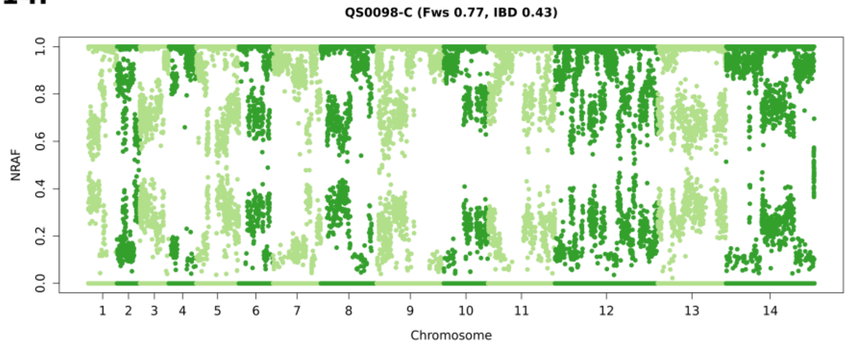

15.

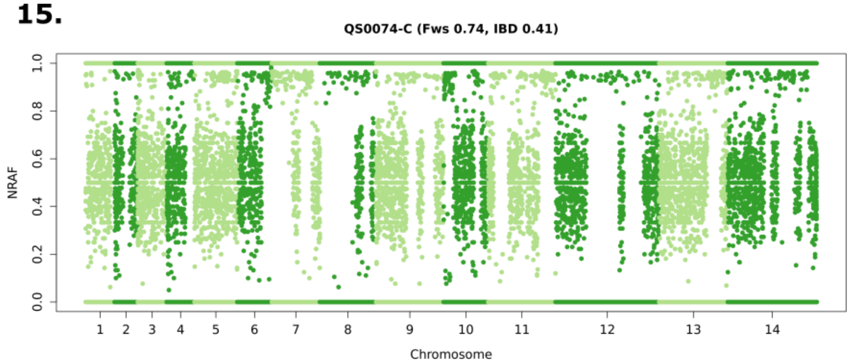

16.

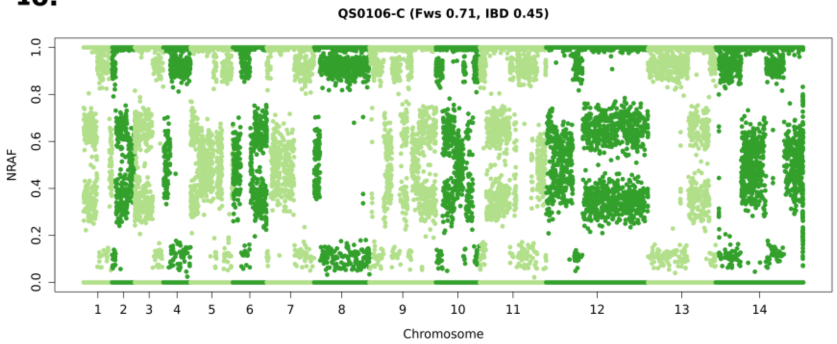

17.

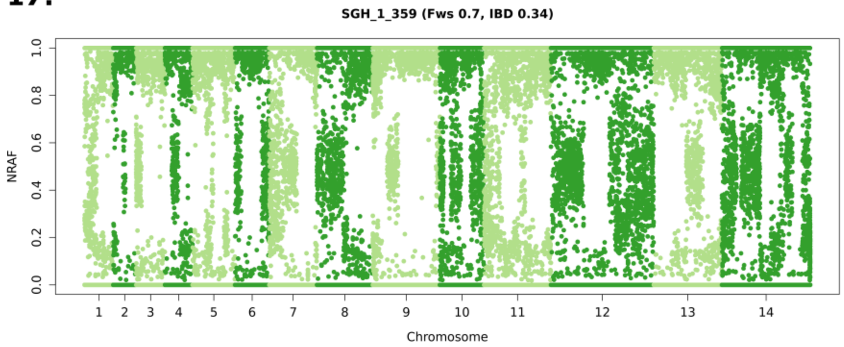

18.

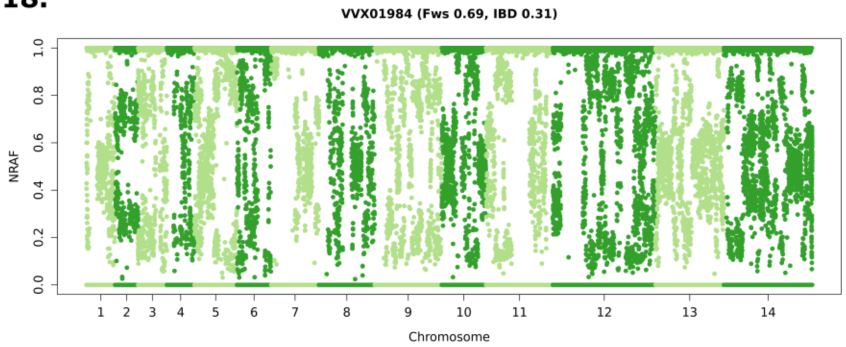

19.

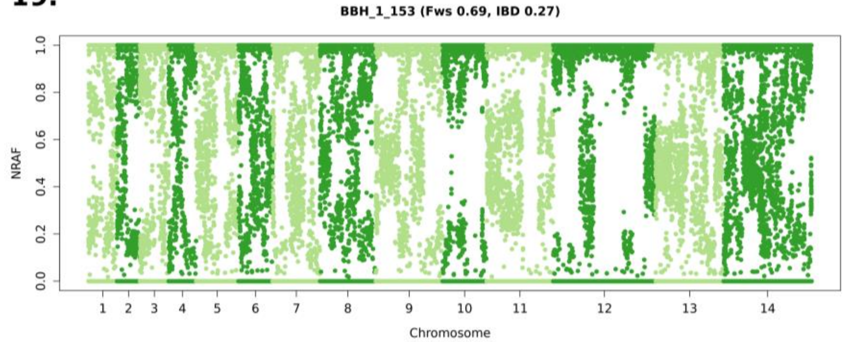

20.

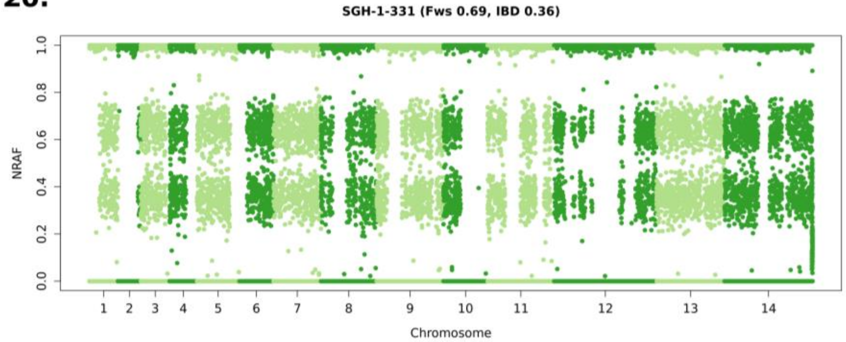

21.

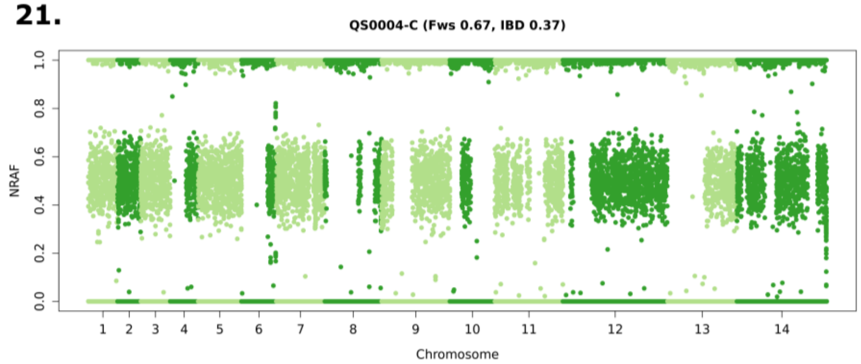

22.

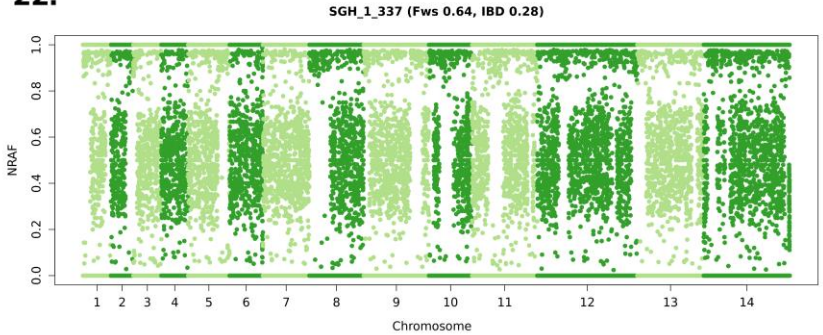

23.

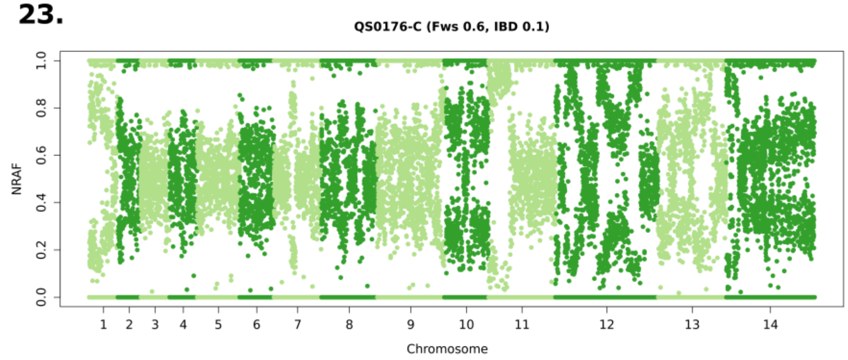

24.

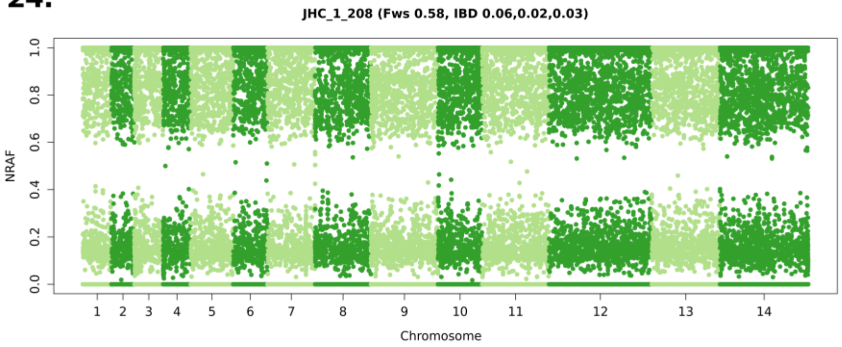

25.

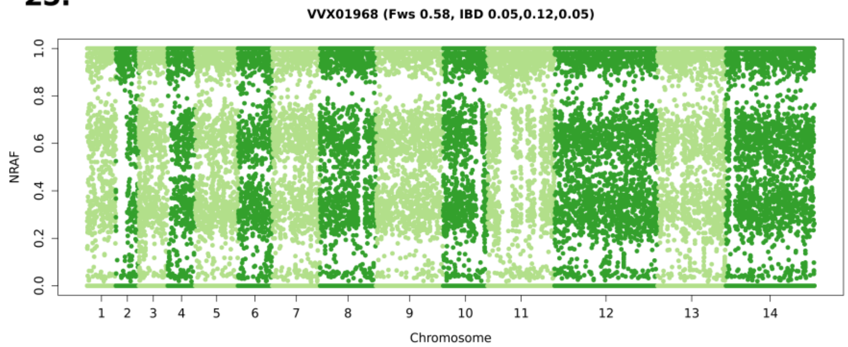

26.

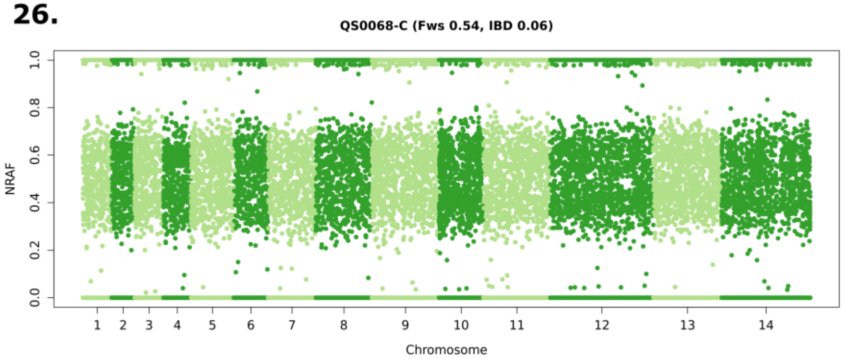

27.

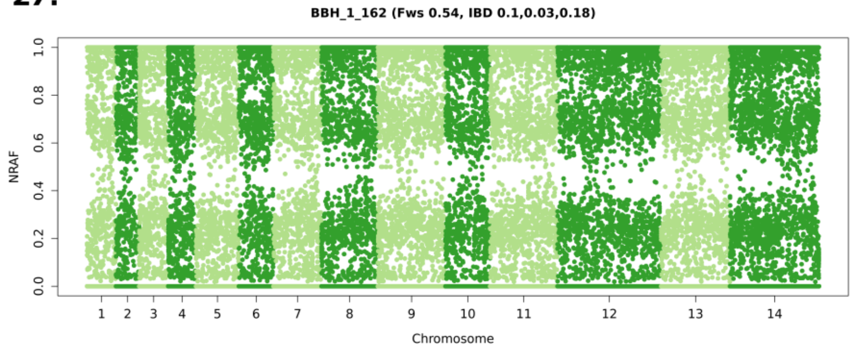

28.

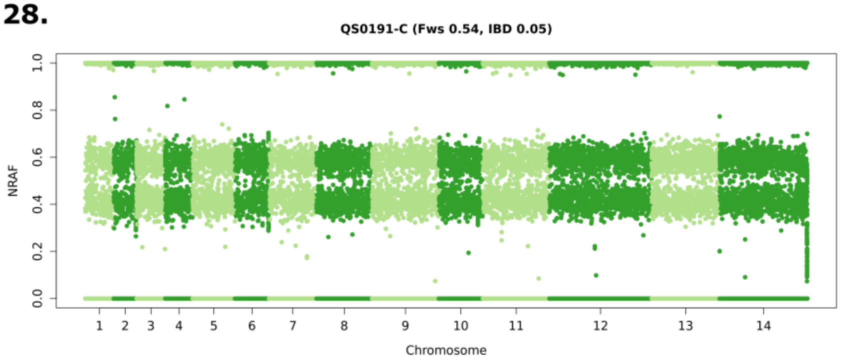

29.

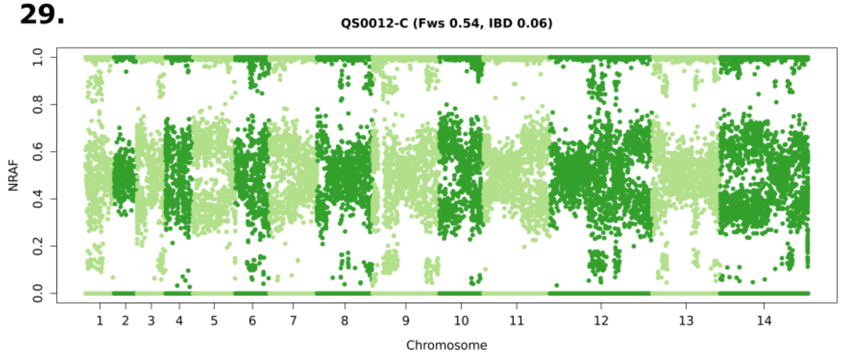

30.

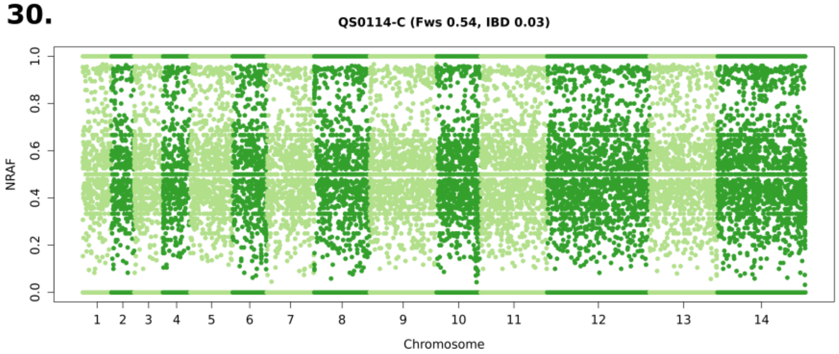

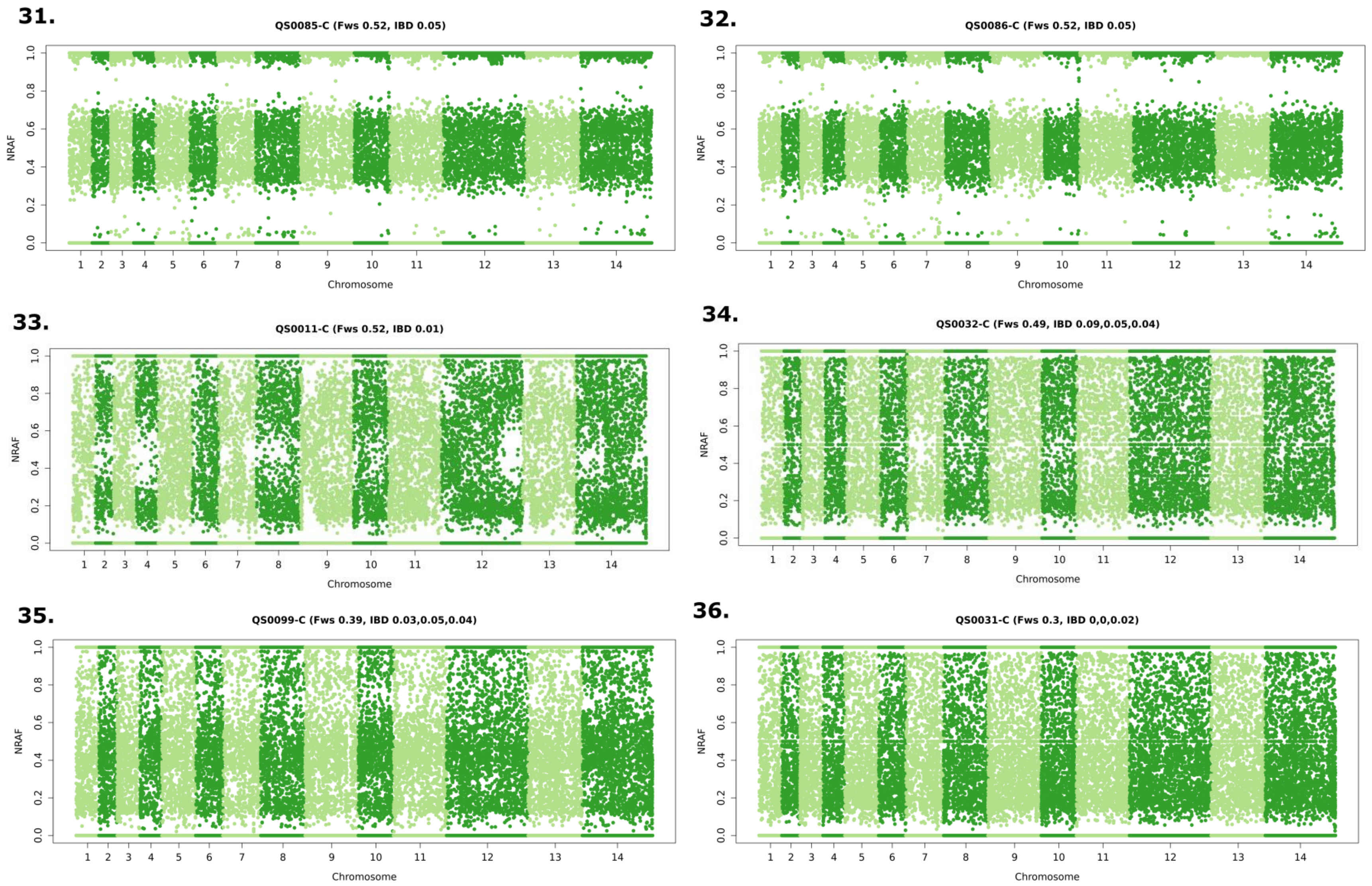

Supplementary Figure 1. Non-reference allele frequency distribution plots for the polyclonal Ethiopian infections.

Samples are sorted by decreasing F_{WS} order. The sample QS0088-C is a clonal infection (F_{WS}=1), provided as a reference point. The samples in panels 5-8, 10-11 and 13-22 all display large segments (≥25%) of relative homology in multiple chromosomes suggestive of half-sibling or greater relatedness between at least two clones within the infection.

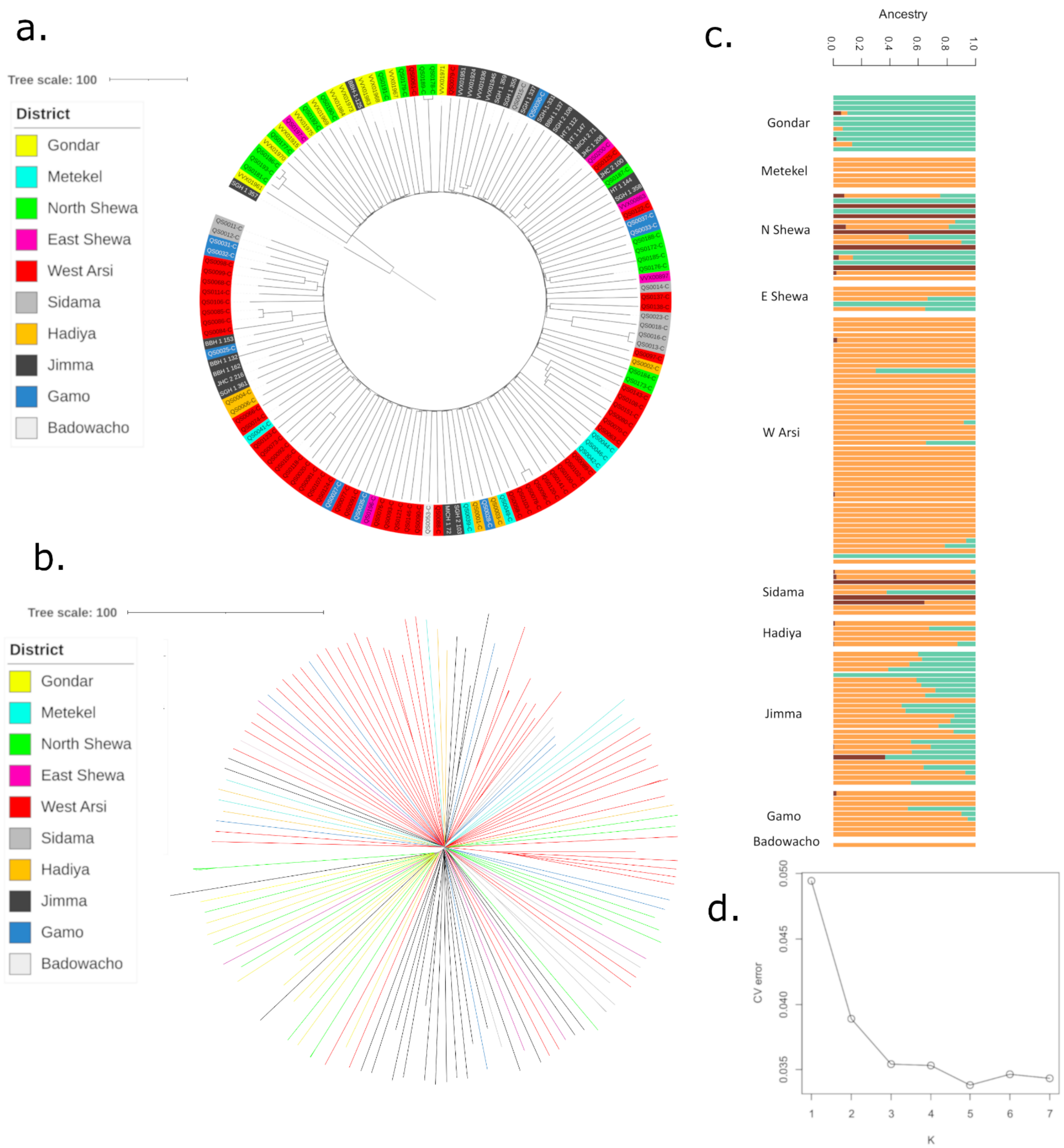

Supplementary Figure 2. *P. vivax* population structure in Ethiopia using measures of IBS.

Panel a) presents a rooted neighbour-joining tree illustrating the relatedness between infections as measured using identity by state (IBS) measures of genetic distance. The tree was rooted at sample SGH-1-357, which displayed the highest divergence from the rest of the population. Panel b) presents an unrooted configuration of the neighbour-joining tree. The neighbour-joining trees are not intended to show historical phylogeny, rather, patterns of relatedness reflecting recent epidemiological events. Panel c) presents a bar plot illustrating the ancestry of each of the infections to 3 sub-populations (i.e., K=3) as defined with ADMIXTURE analysis; K1 in orange, K2 in turquoise

and K3 in brown. Panel d) presents the CV error for the ADMIXTURE analysis. The neighbour-joining trees and bar plot illustrate a large degree of clustering between infections from the northern districts of Gondar and North Shewa, and amongst the infections from Jimma relative to the other sites. The plots are based on data generated using *n*=102 independent monoclonal infections.

Supplementary Table 1. Summary of the Epidemiology at the Ethiopian study sites.

Site (district)	Region	Town(s)	Elevation [b]	Population Size [c]	API [d]
Gondar	Amhara	Maksegnit	2,133	299,969	API >10 & ≤50
Metekel	SNNPR	Guba, Shone	1,909	22,899	API ≥50
North Shewa	Amhara	Shewa Robit	1,280	62,353	API >0 & ≤5
East Shewa	Oromia	Batu, Bishoftu	1,677	80,933	API >0 & ≤5
West Arsi	Oromia	Abosto, Bisha-Gurach, Aje, Ilala, Melka Oda	2,043	218,969	API ≥ 10&<50
Sidama	SNNPR	Hawassa	1,708	324,600	API ≥50
Hadiya	SNNPR [a]	Hadiya	2,106	1,026,824	API ≥ 10 & <50
Jimma	Oromia	Jimma	1,780	203,988	API ≥10 & <50
Gamo	SNNPR	ArbaMinch	1,126	253,112	API ≥50

[a]SNNPR, South Nations Nationalities and People's Republic. [b] Elevation meters above sea level. [c] Population size in 2020 (data from Central Statistics agency). [d] API, Annual Parasite Incidence: number of malaria parasites (all species) per 1,000 population at risk in 2020 (data from National Malaria Elimination Program (NMEP) 2020 malaria stratification plan).

Supplementary Table 2. Spatial trends in *P. vivax* connectivity within and between districts.

	Gondar	Metekel	North Shewa	East Shewa	West Arsi	Sidama	Hadiya	Jimma	Gamo
**Gondar**	0.070	285	385	590	568	658	550	536	690
**Metekel**	0.045	0.079	484	503	554	552	427	362	520
**North Shewa**	0.059	0.044	0.053	320	252	420	370	432	532
**East Shewa**	0.049	0.069	0.053	0.053	142	67	90	195	183
**West Arsi**	0.045	0.071	0.047	0.068	0.069	192	216	318	330
**Sidama**	0.046	0.073	0.044	0.070	0.072	0.096	121	213	147
**Hadiya**	0.043	0.076	0.048	0.067	0.068	0.070	0.067	105	160
**Jimma**	0.049	0.064	0.051	0.063	0.063	0.062	0.064	0.063	165
**Gamo**	0.044	0.071	0.049	0.064	0.069	0.068	0.070	0.064	0.068

The lower triangle presents median identity by descent (IBD) amongst the parasites in the given district combinations. Levels of relatedness are colour-coded as follows; dark red for IBD≥0.09, light red for 0.075≥IBD<0.09, orange for 0.06≥IBD<0.075, yellow for 0.045≥IBD<0.06, and white for IBD<0.045. The upper triangle presents the geographic distance in kilometres between the approximate mid-points of the respective districts. Gondar and North Shewa appear to have the lowest connectivity to other districts, and overall patterns of relatedness do not appear to be fully explained by geographic distance. The data are based on data generated using n=102 independent monoclonal infections.

Supplementary Table 3. Summary of multi-SNP regions with evidence of extended haplotype homozygosity

Test	Test Populations	Signal	Chr	Coordinates	Length	No SNPs	No Genes	Coordinate and function of maximum Rsb Score	Putative genetic Drivers
Rsb	**Ethiopia vs Thailand**	1	1	472655:490006	17351	27	1	472806: Intergenic	Drug-related candidates including PvP01_0110100 (prodrug activation and resistance esterases, putative) in the region, and PvP01_0109300 (chloroquine resistance transporter) in the vicinity.
		2	1	836114:836898	784	22	1	836798: PvP01_0118300	PvP01_0118300 (Lysine specific histone demethylase, putative)
		3	3	424327:424846	519	8	1	424846: PvP01_0309000	PvP01_0309000 (Peptide chain release factor 2, putative)
		4	3	716147:733039	16892	4	5	716147: PvP01_0316400	PvP01_0316400 (Phosphoinositide-binding protein PX1, putative)
		5	4	401500:401685	185	14	1	401622: PvP01_0409900	PvP01_0409900 (acyl-CoA-synthetase, putative)
		6	4	752089:753820	1731	18	1	752636: PvP01_0418200	PvP01_0418200 (Conserved Plasmodium protein, unknown function). Also, a cluster of serine-repeat antigens (SERA) in the vicinity (5'), and PVP01_0418300 (merozoite surface protein 4) and PVP01_0418400 (merozoite surface protein 5) in the vicinity (3').
		7	5	721363:722144	781	7	1	721363: PvP01_0517200	PvP01_0517200 (Zinc finger protein, putative)
		8	6	131431:131475	44	3	1	131431: PvP01_0603400	PvP01_0603400 (Serine/Threonine protein phosphatase 8, putative)

		9	6	272690:286430	13740	7	2	286370/40: PvP01_0606900	PvP01_0606900 (VPS13 domain-containing protein, putative) or PvP01_0606800 (deoxyribose-phosphate aldolase, putative)
		10	7	551200:551443	243	10	1	551376: PvP01_0711200	PvP01_0711200 (tRNA (adenine (58)-N (1))-methyltransferase non-catalytic subunit TRM6, putative)
		11	8	1104527:1106736	220	6	1	1105020: PvP01_0824800	PvP01_0824800 (conserved Plasmodium protein, unkown function)
		12	11	677202:677295	93	3	1	677295: PvP01_1115800	PvP01_1115800 (Conserved Plasmodium protein, unknown function)
		13	11	1423431:1423489	58	3	1	1423431: PvP01_1133300	PvP01_1133300 (Conserved oligomeric Golgi complex subunit 4, putative)
		14	12	788701:789711	1010	35	1	788957: PvP01_1219200	PvP01_1219200 (Hypothetical protein)
		15	12	1420596:1420678	82	3	N/A	1420643: Intergenic	PvP01_1235600 (sulfate transporter) 5' of signal or PvP01_1235700 (exported protein 2, putative) 3' of signal.
		16	13	1140855:1197809	56954	10	21	1140866: PvP01_1326200	PvP01_1326200 (actin-related protein ARP4, putative)
		17	13	1288193:1288469	276	9	1	1288469: PvP01_1330800	PvP01_1330800 (Liver specific protein 1, putative)
Rsb	**Ethiopia vs Indonesia**	2	1	836876:836898	22	2	1	836898: PVP01_0118300	PVP01_0118300 (Lysine specific histone demethylase, putative)
		9**	6	286340:427820	141480	5	30	427733: PvP01_0609500	PvP01_0609500 (ATP synthase-associated protein, putative) or PvP01_0607800 (kelch protein K10, putative)
		14	12	788821:789569	748	18		789486: PvP01_1219200	PvP01_1219200 (Hypothetical protein). Also, cluster of MSP7-like genes upstream.

	17	13	1288004:1288733	729	15	1	1288299: PvP01_1330800	PvP01_1330800 (Liver specific protein 1, putative)
iHS	18	14	3009295:3012815	3520	6	1	3012071: Intergenic	Several Plasmodium exported proteins including PvP01_1470400 (downstream) and PvP01_1470500 (upstream) in the vicinity of the signal.
